# Anisotropic Growth of Centimeter‐Size CsCu_2_I_3_ Single Crystals with Ultra‐Low Trap Density for Aspect‐Ratio‐Dependent Photodetectors

**DOI:** 10.1002/advs.202206417

**Published:** 2023-01-04

**Authors:** Sancan Han, Jiale Quan, Ding Wang, Huijun Li, Xinya Liu, Jingcheng Xu, Yixin Zhang, Ziqing Li, Limin Wu, Xiaosheng Fang

**Affiliations:** ^1^ School of Materials Science and Engineering University of Shanghai for Science and Technology Shanghai 200093 P. R. China; ^2^ Department of Materials Science Institute of Optoelectronics State Key Laboratory of Molecular Engineering of Polymers Fudan University Shanghai 200438 P. R. China; ^3^ College of Chemistry and Chemical Engineering Inner Mongolia University Hohhot 010021 P. R. China

**Keywords:** CsCu_2_I_3_, anisotropic growth, photodetectors, single crystals, trap state density

## Abstract

Low‐dimensional ternary copper iodide metal halide with strong quantum confinement effects has made great progress in optoelectronic fields. However, efficient regulation of anisotropic growth of metal halides single crystal still remains a great challenge. Herein, 2 cm size CsCu_2_I_3_ single crystals with tunable aspect ratio and the trap states (*n*_trap_) as low as 5.38 × 10^9^ cm^−3^ are fabricated by optimized anti‐solvent vapor‐assisted method, in which the growth cycle is shortened by half. Evidenced by real‐time observation and the LaMer growth model, the rapid and anisotropic growth mechanism is ascribed to preferential 1D growth, promoted by high concentration and fast vapor rate. Furthermore, the aspect‐ratio‐dependent optoelectronic performance is observed, the on–off ratio for 2 cm CsCu_2_I_3_ single crystal are enhanced 350 times compared with those of short and thick single crystal, which shows ultrahigh on‐off ratio of 1570, D* of 1.34 × 10^12^ Jones, *R*_*λ*_ of 276.94 mA W^−1^, *t*_rise_ /*t*_decay_ of 0.37 and 1.08 ms, and EQE of 95.53%, which are clearly at very high level among lead‐free perovskite‐based photodetectors. This study not only provides a new strategy for overcoming anisotropic growth limitations of low‐dimensional metal halides, but also paves a way for high‐performance optoelectronic applications.

## Graphical abstract

CsCu_2_I_3_ single crystals with tunable aspect ratio and low trap states are fabricated by optimized antisolvent vapor‐assisted method. The rapid and anisotropic growth is ascribed to preferential 1D growth mechanism, promoted by high concentration and fast vapor rate. CsCu_2_I_3_ single crystals show aspect‐ratio‐dependent optoelectronic performance with the ultrahigh on‐off ratio of 1570.

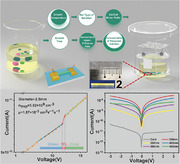

## 1 Introduction

Inorganic lead halide perovskite has been deemed as high promising candidate material in optoelectronic field by many distinguishing features, such as adjustable quantum well distribution, largely tunable direct band gaps, ultra‐long carrier diffusion length, and anomalous defect tolerance.^[^ ^1^, ^2^, ^3^, ^4^ ^]^ However, the toxicity of lead has severely limited its practical applications and commercialization.^[^ ^5^ ^]^ Under such circumstances, environment‐friendly metal cations have been studied to replace Pb^2+^, including Sn^2+^, Bi^3+^, Sb^3+^, and Ag^+^.^[^ ^6^, ^7^, ^8^, ^9^, ^10^ ^]^ There exists still many issues to be solved: 1) the instability against moisture, heat, and light; 2) the non‐ideal optoelectronic properties. Hence, inheriting the excellent optoelectronic properties of Pb‐based perovskite while ensuring the high environmental stability of lead‐free perovskite materials is still a challenge to be overcome at this stage.

CsCu_2_I_3_ regarded as an ideal candidate of novel lead‐free metal halide perovskite due to unique photophysical properties and good stability, has been investigated in the field of photodetection and photoluminescence.^[^ ^10^ ^]^ For example, high‐performance deep ultraviolet photodetector was constructed based on 1D lead‐free halide perovskite CsCu_2_I_3_ film.^[^ ^11^ ^]^ Oriented‐Structured CsCu_2_I_3_ Film were used for high‐resolution X‐ray imaging.^[^ ^12^ ^]^ CsCu_2_I_3_ film‐based UV photodetectors prepared with CuI buffer layers have significantly improved optoelectronic properties.^[^ ^13^ ^]^ High‐performance yellow LEDs based on nontoxic CsCu_2_I_3_ films were successfully developed by using solution‐processed.^[^ ^14^ ^]^ However, the grain boundaries are highly vulnerable to moisture and especially unstable, which leads to low carrier mobility and undesired charge recombination. Recently, the single crystal perovskites without the grain boundaries exhibit enhanced properties in terms of trap density and carrier mobility characteristics.^[^ ^15^ ^]^ The trap density of single crystals was reported to be between 10^9^–10^11^ cm^−3^, which is ≈5 orders of magnitude lower than the trap density of polycrystalline films, resulting in ultralong charge carrier diffusion lengths of several tens to hundreds of micrometers.^[^ ^16^ ^]^ Hence, it has become a consensus that perovskite single crystals are significant for the development of high‐performance and stable optoelectronic devices. In addition, 1D nanostructure with high aspect ratio and well‐defined morphology is perfect for photodetection due to the efficient transport of carriers along the longitudinal direction.^[^ ^17^ ^]^ For instance, facet‐dependent and broadband photodetector was built up by highly stable CsCu_2_I_3_ single crystal.^[^ ^18^ ^]^ 1D CsCu_2_I_3_ single crystals for white‐light emitting diodes and UV photodetection were constructed with a photoluminescence quantum yield (PLQY) of 50.4% and a UV response time of 50.4 ms.^[^ ^19^ ^]^ However, these above single crystals were too short or too thick in size, the search for efficient methods to achieve single crystal with desirable size and morphology and thus ideal optoelectronic performance is of vital to expand the potential of applications.

These properties of single crystals, such as size, morphology, and quality, impart them with unique optoelectronic property. Therefore, well understanding and precisely controlling the growth of perovskites single crystal could further obtain ideal optical and electronic performance.^[^ ^20^ ^]^ To date, much progress has been made in improving the quality of single crystals and the controlled preparation of perovskite single crystals of different size and morphology.^[^ ^21^, ^22^, ^23^ ^]^ Little research has been done in this area for CsCu_2_I_3_ single crystals due to its fast nucleation, hence, systematic study about how related growth parameters affect nucleation, growth, size and morphology evolution remains insufficient. In the work, ultra‐long centimeter‐size CsCu_2_I_3_ single crystal was prepared by an optimized anti‐solvent vapor‐assisted method, in which the growth cycle is shortened by half in comparison with previous reports. Meanwhile, the single crystal length (0.59–18.8 mm) and diameter (72–2590 µm) can be easily and efficiently regulated. The real‐time observation of CsCu_2_I_3_ crystal growth evidenced three stages in the LaMer growth model: nucleation aggregation (stage I), initial three‐dimensional (3D) growth (stage II) and preferential 1D growth (stage III), which can clearly explain the formation of ultra‐long centimeter‐size CsCu_2_I_3_ single crystal. The optimized antisolvent vapor‐assisted method in this study provided a more suitable growth environment to promote the C axis ([001] crystal direction) growth. The calculated *n*_trap_ of CsCu_2_I_3_ single crystals are 2.77 × 10^11^ cm^−3^ (diameter = 200 µm), 3.17 × 10^10^ cm^−3^ (diameter = 1 mm), and 5.38 × 10^9^ cm^−3^ (diameter = 2.5 mm), which strongly verify the formation of high quality CsCu_2_I_3_ single crystals. The aspect‐ratio‐dependent photoelectric performance was observed, the on–off ratio for 2 cm CsCu_2_I_3_ single crystal are enhanced 350 times compared with those of short and thick single crystal, which shows high on‐off ratio of 1570, D* of 1.34 × 10^12^ Jones, *R*_*λ*_ of 276.94 mA W^−1^, *t*_rise_ /*t*_decay_ of 0.37 and 1.08 ms, and EQE of 95.53%.

## 2 Results and Discussion

### 2.1 Characterization of High quality and Ultra‐Long Centimeter‐Sized Single Crystal

**Figure 1:**
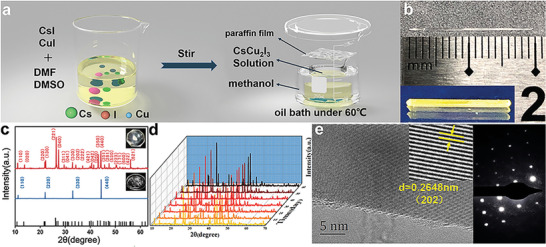
a) Schematic procedures for the synthesis of CsCu_2_I_3_ Single crystals. b) Optical photograph of millimeter‐sized CsCu_2_I_3_ ultra‐long single crystals, and the inset is the photograph of single crystals under UV illumination. c) XRD patterns of CsCu_2_I_3_ SCs and its powder (insets are corresponding photographs). d) The time dependent XRD patterns of the CsCu_2_I_3_ single crystal remained essentially constant with gradually increasing time. e) High‐resolution TEM image of CsCu_2_I_3_, the inset is the enlarged section, and right side of the image is the corresponding fast Fourier transform (FFT) image of CsCu_2_I_3_.

A schematic diagram of the single crystal growth process is shown in **Figure** **1**a. The supersaturated optimized growth interval is crucial to achieve the fast and anisotropic growth of high‐quality centimeter‐sized single crystals.^[^ ^24^, ^25^ ^]^ Figure 1b presents the photograph of freshly as‐grown single crystals, showing that ultra‐long (≈2 cm), high‐aspect‐ratio CsCu_2_I_3_ single crystal with the diameter of 200 µm is successfully synthesized for the first time, which shines bright yellow under the illumination of 360 nm UV light (Figure 1b inset). As shown in Figure S1 (Supporting Information), high transparency in sunlight evidenced the high quality of CsCu_2_I_3_ single crystal. The crystal structure of the CsCu_2_I_3_ was confirmed by X‐ray diffraction (XRD) analysis (Figure 1c). The strong diffraction peaks of the CsCu_2_I_3_ powders at 11.02°, 21.85°,26.34, 32.88°,44.23°, and 56.08° are assigned to the (110), (220), (221), (330), (440), and (621) planes, confirming the orthorhombic CsCu_2_I_3_ crystal structure in space group Cmcm (PDF#45‐0076). In addition, the XRD pattern of CsCu_2_I_3_ single crystal shows four dominant peaks at 10.83°, 21.45°,32.82°, and 44.62° corresponding to the (110), (220), (330), and (440) planes, respectively, evidencing the formation of high‐ crystalline quality CsCu_2_I_3_ single crystal. The stability under open air atmosphere (at least 70%RH moisture) of CsCu_2_I_3_ single crystal was further investigated by performing a time‐dependent XRD measurement. As shown in Figure 1d, the diffraction peaks did not change significantly within 60 days, which indicates that CsCu_2_I_3_ single crystal maintains high phase purity and crystalline quality. In Figure S2 (Supporting Information), it can be clearly seen that the surface of CsCu_2_I_3_ single crystal is flat and smooth, revealing high crystalline quality and phase purity for CsCu_2_I_3_ single crystal, which is consistent with the XRD results. Figure S3 (Supporting Information) displays the focused ion beam (FIB) slices with a width of 5.135 µm, a height of 4.902 µm, and a thickness of 100 nm to conduct transmission electron microscope (TEM). The visible crystal lattice fringe with lattice spacing of 0.264 nm, which is corresponding to (202) crystal plane, is showed in Figure 1e, and the corresponding fast Fourier transform (FFT) image confirms the formation of the high‐quality CsCu_2_I_3_ single crystal. In Figure S4 (Supporting Information), the elemental mapping images indicate that Cs, Cu, and I were homogeneously distributed in the single crystal slices. The survey X‐ray photoelectron spectroscopy (XPS) as shown in Figure S5 (Supporting Information) shows the peaks of the constituent elements C 1s, Cs 3d, Cu 2p, and I 3d, which are in good agreement with the reported literature.^[^ ^18^, ^19^ ^]^ The element ratio of Cs:Cu:I is calculated to be 10.08:20.51:27.07, which is close to the nominal ratio of CsCu_2_I_3_.

### 2.2 Anisotropic Growth of Single Crystal

**Figure 2:**
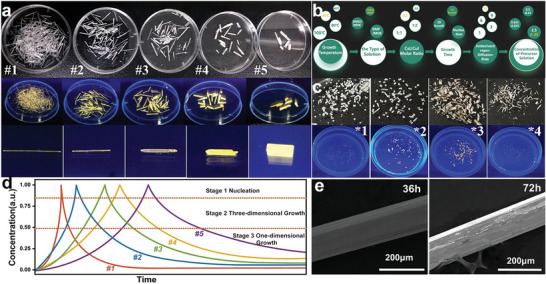
a) The evolution of the single crystal size is influenced by the diffusion rate of antisolvent vapor in precursor solution. Top: the photographs of freshly as‐grown single crystals. Middle and bottom: the photographs of as‐grown single crystals under UV illumination. b) Summary diagram of CsCu_2_I_3_ single crystal growth parameters regulation. c) Effect of growth temperature on the quality of single crystals. Top: Photographs of single crystals grown at different temperatures; Bottom: Photographs of single crystals under 365 nm UV light irradiation. d) LaMer model for the growth of CsCu_2_I_3_ single crystal at different diffusion rate of antisolvent vapor. (e) SEM images of CsCu_2_I_3_ single crystals at different growth times.

In order to obtain the optimized growth technics of high‐quality CsCu_2_I_3_ single crystal, different growth parameters regulations are showed in **Figure** **2**. Figure 2a shows high‐quality CsCu_2_I_3_ with different aspect ratios, which is influenced by the rate of entry of the antisolvent vapor into the precursor solution (#1, #2, #3, #4, #5 samples are referred to 12 small holes, 9 small holes, 6 small holes, 3 small holes, 1 small hole on the paraffin film). Figure S6 and Table S1 (Supporting Information) summarize the dimensions of the CsCu_2_I_3_ single crystal prepared under different diffusion rate of antisolvent vapor in precursor solution. The mean value and standard deviation of the dimensions are calculated based on some 30 crystals. It can be clearly observed that the modulation of the antisolvent diffusion rate allows efficient adjustment of the length of CsCu_2_I_3_ single crystals in the range of 4.82–18.8 mm and the diameter in the range of 158–2590 µm. With the increasing antisolvent vapor entering the precursor solution increases, a decrease in diameter and a subsequent increase in length are observed. Figure 2b shows the summary diagram of CsCu_2_I_3_ single crystal growth parameters regulation. In Figure S7 and Table S2 (Supporting Information), the single crystals (the length: 0.59–15.62 mm, the diameter: 72–353 µm) are obtained by varying the concentration of the precursor solution. Figure S8 (Supporting Information) demonstrates that the higher the concentration of the precursor solution, the longer of single crystals tends to be. Figure 2c shows the effect of growth temperature on the quality of single crystals (*1: 40, *2: 60, *3: 80, and *4: 100 °C). When the temperature is 40 °C, the single crystal grows slowly (4–5 days) and the grown single crystal has low transparency and lusterless surface, which can be ascribed to the insufficient growth momentum for antisolvent diffusion under lower temperature. When the temperature is too high to reach 80 or 100 °C, single crystal in the Cu^+^ cations is easy to be oxidized to Cu^2+^ cations,^[^ ^19^, ^25^ ^]^ hence black powders deposit on single crystal surface. Only when the growth temperature reaches to 60 °C, the single crystal with high transparency and surface gloss are obtained, meanwhile, the luminescence intensity under UV light irradiation is high. The powder XRD measurements were further carried out to investigate the structural variation of different CsCu_2_I_3_ single crystal, as presented in Figures S9–S13 (Supporting Information). The powder XRD patterns of all the CsCu2I3 single crystals are in good agreement with the standard card (PDF#45‐0076; Space group, Cmcm). In combination with Figures S14–S17 (Supporting Information), the CsCu_2_I_3_ single crystals prepared with a molar ratio of CsI:CuI = 3:2 at 60 °C for 36 h grown in DMF/DMSO owns the highest crystalline, which is consistent with the above XRD results.

In situ observation of CsCu_2_I_3_ single crystals (#1, 12 small holes) was carried out to gain a deeper understanding of the growth mechanism. The LaMer growth model in Figure 2d shows that there are three stages during CsCu_2_I_3_ crystal growth process: nucleation aggregation (stage I), initial 3D growth (stage II) and preferential 1D growth (stage III). In stage I, as the antisolvent vapor gradually enters the precursor solution, a nucleation barrier is gradually reached due to solvent exchange. The CsCu_2_I_3_ molecules aggregate and combined through van der Waals interactions. During stage II, growth along the crystal length, width and height directions occurs simultaneously, resulting in some fine ellipsoidal seed crystals with diameter of ≈200 µm (Figure S18, Supporting Information). In stage III, the diameter of the single crystal remained almost constant at ≈200 µm when the growth time is longer than 1.5 h, CsCu_2_I_3_ stacks preferentially along the length direction and gradually forms long single crystals. The duration of stages II and III are the key factors for the growth of centimeter‐size CsCu_2_I_3_ single crystals,^[^ ^26^, ^27^, ^28^ ^]^ hence, the controlled antisolvent vapor diffusion has an absolutely dominant effect on the growth process. When the rate of antisolvent vapor entering the precursor solution increases (from #5 to #1), the nucleation threshold reached more quickly, which leads to the long duration time of the stages II and III, resulting in the formation of centimeter‐size CsCu_2_I_3_ single crystal. In Figure 2e and Figure S19 (Supporting Information), it can be clearly observed that single crystal shown a mature crystal structure with the growth time of 36 h, however, the surface impurities of the single crystal have a tendency to increase when the growth is up to 72 h, which can be explained the increasing probability of nonstoichiometric CsCu_2_I_3_ dendrites or impurities aggregating on the single crystal surface.^[^ ^25^, ^29^ ^]^ In our study, the fast antisolvent diffusion leads to rapid nucleation and aggregation of crystals, which reduces the duration time of the nucleation aggregation (stage I), in turns increasing those of stages II and III, which is beneficial to the formation of the ultra‐long CsCu_2_I_3_ single crystal. Furthermore, rapid formation of high‐quality CsCu_2_I_3_ single crystals happens due to the longer optimized growth regions in the high precursor solution concentration.

### 2.3 Tunable Optoelectronic Properties

**Figure 3:**
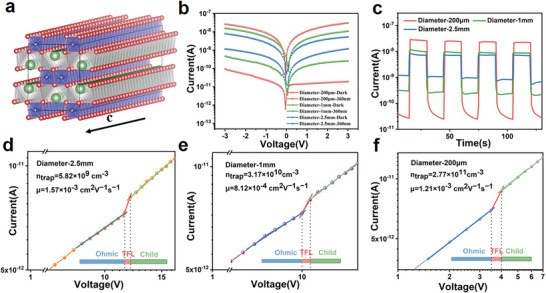
a) 1D crystal structure of CsCu_2_I_3_ single crystal: Perspective view of crystal structure along [001] direction (*c*‐axis); b,c) *I–V* and *I–T* curves of CsCu_2_I_3_ single crystals with different diameters under dark and 360 nm illumination at 3 V bias. d) Diameter‐2.5 mm, e) diameter‐1 mm, and f) diameter‐200 µm CsCu_2_I_3_ Single crystal measured using the SCLC method.

In order to give insight into the reason for the preferential growth of the ultra‐long CsCu_2_I_3_ single crystal in the length direction (defined as *c*‐axis direction), its 1D crystal structure and 1D electronic structure are discussed in **Figure** **3** and figure S20 (Supporting Information). CsCu_2_I_3_ belongs to the orthorhombic system with the space group of Cmcm (a = 10.93 Å, b = 13.90 Å, c = 5.91 Å, and *α* =*β* = *γ* = 90°). The [CuI_4_]^3−^ tetrahedra form a double chain by shared edges, and the unit structure of the double chain is the [Cu_2_I_6_]^4−^ octahedra, as shown in Figure 3a and Figure S20a (Supporting Information).^[^ ^11^, ^30^ ^]^ Double chain (core) are surrounded and isolated by rows of Cs^+^ ions (shell), similar to the core–shell structure. The chains of edge‐sharing [Cu_2_I_6_]^4−^ octahedra as the core and Cs^+^ ions wrapped in the outer layer form 1D crystal structure along [001] direction, therefore CsCu_2_I_3_ single crystals can preferentially grow along the *c*‐axis ([001] crystal direction), forming high aspect ratio CsCu_2_I_3_ single crystals.^[^ ^13^, ^18^ ^]^

The density functional theory (DFT) calculation based on the primitive cell is conducted to characterize the electronic structure of CsCu_2_I_3_. As shown in Figure S20b (Supporting Information), high dispersion bands near the valence and conduction edges illustrate the excellent carrier transport properties of the copper‐based perovskite material.^[^ ^18^ ^]^ The total and partial density of states (DOS) are shown in Figure S20c (Supporting Information), and it can be clearly seen that the CBM of CsCu_2_I_3_ is mainly dominated by the Cu 4s and I 5p orbitals, and the VBM is mainly dominated by the Cu 3d and I 5p orbitals. The Cs^+^ cation has little effect on the CBM and VBM, and it may act as the quasi‐isolated shell for chains of edge‐sharing [Cu_2_I_6_]^4−^ octahedra to provide efficient light emissions.^[^ ^19^ ^]^ Above all, it is speculated that photogenerated electrons are localized in the twofold chain created by [CuI_4_] tetrahedra, which facilitates directional charge transport and exciton diffusion. In Figure S21 (Supporting Information), the photocurrent of *c*‐axis direction in CsCu_2_I_3_ single crystal is higher than that of *a*‐axis direction under 360 nm illumination, the switch ratio of *c*‐axis direction in CsCu_2_I_3_ single crystal is enhanced by 18 times. The phenomenon can be explained by that photogenerated electrons are localized in the twofold chain created by [CuI_4_] tetrahedra, leading to the fast transport mobility among the chain, which is parallel to [001] (*c*‐axis) direction, which is consistent with DFT results. Furthermore, the CsCu_2_I_3_ single crystals with different aspect ratio show tunable optoelectronic performance in Figure 3b, displaying the symmetric dark‐current and photocurrent under the illumination of 360 nm. It can be clearly observed that the dark current of the single crystal with 200 µm diameter (1.93 × 10−11 A) is significantly lower than that of the single crystals with 1 mm (2.58 × 10−10 A) and 2.5 mm diameter (1.18 × 10−9 A), while the photocurrent of the single crystal with 200 µm diameter is significantly higher than that of the single crystals with 1 and 2.5 mm diameter. As shown in Figure 3c, the on–off cycle is stable without notable decay, the on‐off ratio for the single crystal with 200 µm diameter of 1570, which is 350 times higher than that of the single crystal with 2.5 mm diameter. The above results indicate that the optoelectronic performance can be adjustable by tunable aspect ratio of CsCu_2_I_3_ single crystals.

In order to derive the densities of trap states (*n*_trap_) of CsCu_2_I_3_ single crystals, we performed space‐charge‐limited current (SCLC) tests using the Ag/CsCu_2_I_3_/Ag device structure, which can be considered as hole‐only device according to the reported literature.^[^ ^31^, ^32^ ^]^ The dark current‐voltage (*I–V*) curves of CsCu_2_I_3_ single crystals with diameters of 200 µm, 1, and 2.5 mm were measured, respectively. The dark *I–V* curve can be divided into three regions, which are a linear ohmic region (blue), the trap filled region (red), and the trap free Child's region (green), as shown in Figure 3d–f. From the second trap filled region (green), the trap density is calculated using the equations:

(1) $$n_{\mathrm{trap}}=\frac{2{V\;}_{TFL}{\varepsilon \varepsilon }_{0}}{eL^{2}}\;$$

where *e* is the elementary charge, *L* is the thickness of the CsCu_2_I_3_ single crystal, *ε* = 28.6 is the relative dielectric constant of the CsCu_2_I_3_ single crystal,^[^ ^19^ ^]^ and *ε*_0_ is the vacuum permittivity. The calculated *n*_trap_ of CsCu_2_I_3_ single crystals are 2.77 × 10^11^ cm^−3^ (diameter = 200 µm), 3.17 × 10^10^ cm^−3^ (diameter = 1 mm), and 0.538 × 10^10^ cm^−3^ (diameter = 2.5 mm), respectively, which strongly verify the formation of high quality and centimeter‐size CsCu_2_I_3_ single crystals. The SCLC model are established at the higher bias of the Child's region (green), the dark current can be determined by the following Mott–Gurney law:

(2) $$J_{D}=\frac{9{\varepsilon \varepsilon }_{0}\mu V_{b}^{2}}{8L^{3}}\;$$

where *J*_D_ is the dark current density, and *V*_b_ is the applied voltage.^[^ ^32^ ^]^ The carrier mobility µ of CsCu_2_I_3_ single crystals with different diameters are calculated to be 1.21 × 10^−3^ cm^2^ V^−1^s^−1^ (diameter = 200 µm), 7.16 × 10^−4^ cm^2^ V^−1^s^−1^ (diameter = 1 mm) and 1.34 × 10^−2^ cm^2^ V^−1^s^−1^(diameter = 2.5 mm), respectively. The relatively lower carrier mobility contributes to the low dark current, which is beneficial to the high‐performance photodetector.

### 2.4 Tunable Optical Performance

**Figure 4:**
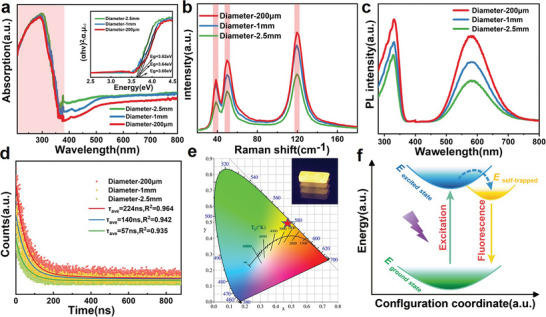
a) Absorption spectrum and corresponding Tauc plot (inset) of the CsCu_2_I_3_ single crystals of different sizes affected by the antisolvent diffusion rate. b) Raman scattering spectra of CsCu_2_I_3_ single crystals of different sizes affected by the antisolvent diffusion rate under ambient conditions. c) PL and PLE spectra of CsCu_2_I_3_ single crystals of different sizes affected by the antisolvent diffusion rate. d)Time‐resolved PL decay and fitting curve for different diameters CsCu_2_I_3_ single crystals e) CIE color coordinates under UV irradiation. The inset is the photograph of single crystal luminescence under UV irradiation. f) Configuration coordinate diagram for the STEs dynamic mechanism of the CsCu_2_I_3_.

The photophysical properties of the CsCu_2_I_3_ single crystals of different sizes are studied in **Figure** **4**. Figure 4a demonstrates optical absorption spectra of CsCu_2_I_3_ single crystals of different sizes, and they both shows obvious absorption in UV region with the wide calculated band gap of 3.62 eV (Diameter = 2.5 mm), 3.64 eV (Diameter = 1 mm) and 3.68 eV (Diameter = 200 µm), respectively (the inset of Figure 4a), which is consistent with the previously reported value.^[^ ^13^, ^14^ ^]^ Figure 4b showing the Raman scattering spectra of CsCu_2_I_3_ single crystals of different aspect ratio under ambient conditions. The characteristic Raman peaks of CsCu_2_I_3_ single crystals are basically the same, and three strong peaks can be observed at 40, 50, and 123 cm^−1^. The strongly polarized Raman spectra indicate the high crystallinity of the CsCu_2_I_3_ single crystal, especially the ultra‐long single crystal with a diameter of 200 µm.^[^ ^33^ ^]^ The photoluminescence excitation (PL) spectrum in Figure 4c shows that CsCu2I3 single crystals with different aspect ratios displayed good optical feature and it sent out yellow emission at ≈580 nm, and 200 µm diameter single crystal displayed the strongest emission. In order to gain further insight into the carrier behavior, transient‐state PL characterization was performed on CsCu_2_I_3_ single crystals with different aspect ratios. As shown in Figure 4d, the PL decay curves of three CsCu_2_I_3_ single crystals can be well fitted with bi‐exponential decay model, in which the average PL lifetime for CsCu_2_I_3_ single crystals was 224 ns (diameter = 200 µm), 140 ns (diameter = 1 mm), and 57 ns (diameter = 2.5 mm), respectively. The CsCu_2_I_3_ single crystals with 200 µm diameter own relatively longer PL lifetime of 224 ns than that of other single crystals, which could facilitate carrier separation, resulting in the enhanced optoelectronic properties.^[^ ^24^ ^]^ The inset of Figure 4e displays a yellow emission photograph of the CsCu_2_I_3_ single crystal, the Commission International de l'Eclairage (CIE) color coordinates of the single crystal under UV irradiation are shown in Figure 4e. The collected yellow emission corresponds to CIE color coordinates of (0.47,0.49), which is a typical yellow region. Figure 4f depicts a plausible PL mechanism of the CsCu_2_I_3_. The incident UV light is absorbed by CsCu_2_I_3_ single crystal, the electrons in the valence band is excited to the excitation state manifold. During relaxation, the free electrons subsequently reorganize into the lower energy self‐ trapped excited state via an ultrafast excited state. Thus, the trapped electrons combine with holes to produce broadband emission with a large Stokes shift.^[^ ^9^, ^14^ ^]^

### 2.5 Application in Photodetector

**Figure 5:**
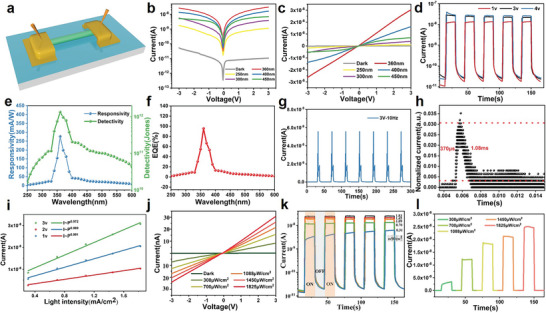
a) Schematic view of the device. b,c) Semilogarithmic *I–V* curves and *I–V* curves under dark and illumination. d) *I–T* curves under 360 nm illumination with different bias voltages. e) Calculated responsivity and detectivity curves of 250–600 nm at 3 V bias. f) Calculated EQE curve of 250–600 nm at 3 V. g) Photoresponse to 10 Hz 360 nm laser pulse at 3 V bias. h) Estimated rise and decay time from single pulse response curve. i) Photocurrent as a function of photodensity and the corresponding power law fitting curves under 360 nm illumination at 3 V, 2 V, 1 V bias, respectively. Light intensity‐dependent *I–V* curves j) and *I–T* curves kl) under 360 nm illumination.

Ultra‐long centimeter‐size CsCu_2_I_3_ single crystals with 200 µm diameter photodetector was constructed to systematically investigate the optoelectronic performances in **Figure** **5**a and Figure S22 (Supporting Information). Figure 5b exhibits the semilogarithmic *I‐*‐*V* curves under the dark and illumination condition. The nonlinear characteristics of *I‐*‐*V* curves evidence the formation of Schottky contacts with Ag electrodes, as shown in Figure 5c. Figure 5d illustrates the *I‐*‐*T* curves of the PD under 360 nm illumination at various voltage, indicating the repeatable photoresponse of the photodetector. The device exhibits a dark current (1.93 × 10^−11^ A) and a markedly enhanced photocurrent (3.03 × 10^−8^ A) with on‐off ratio of 1570 at 3 V, which is much higher than most photodetectors based on all‐inorganic lead‐free metal halide and is even comparable to photodetectors based on all‐inorganic lead‐based metal halide.^[^ ^16^, ^34^, ^35^ ^]^

The responsivity (*R*_*λ*_), detectivity (D*), and external quantum efficiency (EQE) are introduced to determine the optoelectronic performance of the photodetectors,^[^ ^7^ ^]^ which are calculated using the relation as follows:

(3) $$R_{\lambda }=\frac{I_{\mathrm{ph}}-I_{\mathrm{dark}}}{p_{\lambda }S}\;$$

(4) $$D^{*}=\frac{R_{\lambda }}{{\left(2eI_{\mathrm{dark}}/S\right)}^{1/2}}\;$$

(5) $$EQE\;=\frac{hc}{e}\;\frac{R_{\lambda }}{\lambda }$$

in which *h*, *e*, and *c* are the plank constant, elementary charge, and the light velocity, respectively. Meanwhile, *λ*, *P*_*λ*_, *I*_ph_, *I*_dark_, and *S* represent the illumination wavelength, light power density, photocurrent, dark current, and effective illumination area of the photodetector, respectively. In addition, the UV light intensity is 1088µW cm^−2^. As shown in Figure 5e,f, the device shows a responsivity of 276.94 mA W^–1^ and a specific detectivity of 1.34 × 10^12^ Jones at 3 V bias voltage under 360 nm illumination, and shows outstanding optoelectronic conversion efficiency with EQE up to 95.53%, which are clearly at very high level among lead‐free perovskite‐based photodetectors.^[^ ^34^, ^35^, ^36^ ^]^ The time‐resolved pulse response curve of the photodetector was obtained by using 355 nm laser pulses with 10 Hz frequency, highly stable, repeatable photoresponse is observed in Figure 5g. As seen in Figure 5h, the rise and decay time can be estimated to be ≈370 µs and ≈1.08 ms, respectively, which is much faster than most photodetectors.

The relationship of the photocurrent with light intensity is fitted by a power law, *I*_ph_ = AP^*θ*^, in which A is a constant and the exponent *θ* determines the response of the photocurrent to irradiation intensity.^[^ ^7^ ^]^ Figure 5i illustrates the curve fitted, and the value of *θ* are 0.99, 0.98, and 0.97 at the bias of 1.0, 2.0, and 3.0 V, respectively, evidencing the near‐linear dependence of the photodetector. The photocurrent is shown as the function of photodensity at a fixing bias voltage and light wavelength, owing to proportional relationship between the photoinduced carrier and the absorbed photon flux. Figure 5j displays nonlinear *I‐* *V* curves of the CsCu_2_I_3_ single crystal photodetector under 360 nm illumination and varying light intensities. Figure 5k–l display the photoresponse decreases at 3 V bias under 360 nm illumination with the decreasing of photodensity from 1825 to 308 µW cm^−2^. Table S3 (Supporting Information) summarizes the photodetector performance based on copper‐based perovskites and other typical all‐inorganic metal halide perovskites. It can be seen that the performance of ultra‐high aspect ratio single crystal is in the leading position compared to lead‐free halide perovskites‐based devices.

## 3 Conclusions

In summary, high‐quality centimeter‐scale CsCu_2_I_3_ single crystals with different aspect ratios were successfully obtained by exploring and optimizing the growth parameters. The single crystal length (0.59–18.8 mm), diameter (72–2590 µm) can be easily and efficiently regulated, in which the controlled antisolvent vapor diffusion and precursor solution concentration plays a dominant role in the size control of single crystals. Appropriate growth time and molar ratio of reagents will reduce the probability of the appearance of non‐stoichiometric CsCu_2_I_3_ or impurities on the surface. It is speculated that photogenerated electrons are localized in the twofold chain created by [CuI_4_] tetrahedra, which facilitate directional charge transport and exciton diffusion. The superior photodetection performance in the single crystal length direction over the transverse direction provides an argument for this speculation. The single crystal has the low densities of trap states (*n*_trap_) and the low dark current and longer PL lifetime, which is beneficial to high performance photodetector. The work is of great significance for the lead‐free based perovskite to achieve desirable single crystal and optoelectronic performance.

## Conflict of Interest

The authors declare no conflict of interest.

## Supporting information

Supporting Information

## Data Availability

The data that support the findings of this study are available in the supplementary material of this article.

## References

[1] P. Zhang , Y. Hua , Y. Xu , Q. Sun , X. Li , F. Cui , L. Liu , Y. Bi , G. Zhang , X. Tao , Adv. Mater. 2022, 34, 2106562. 10.1002/adma.202106562 35062044

[2] M. Z. Rahaman , S. P. Ge , C.h. H. Lin , Y. M. Cui , T. Wu , Small Struct. 2021, 2, 2000062.

[3] Y. Song , L. Li , M. Hao , W. Bi , A. Wang , Y. Kang , H. Li , X. Li , Y. Fang , D. Yang , Q. Dong , Adv. Mater. 2021, 33, 2103078. 10.1002/adma.202103078 34637161

[4] Z. Tan , Y. Wu , H. Hong , J. Yin , J. Zhang , L. Lin , M. Wang , X. Sun , L. Sun , Y. Huang , K. Liu , Z. Liu , H. Peng , J. Am. Chem. Soc. 2016, 138, 16612. 27966926 10.1021/jacs.6b11683

[5] Z. Xiao , Z. Song , Y. Yan , Adv. Mater. 2019, 31, 1803792. 10.1002/adma.201803792 30680809

[6] Z. Ma , Z. Shi , D. Yang , F. Zhang , S. Li , L. Wang , D. Wu , Y. Zhang , G. Na , L. Zhang , X. Li , Y. Zhang , C. Shan , ACS Energy Lett. 2019, 5, 385.

[7] Z. Q. Li , X. Liu , C. Zuo , W. Yang , X. S. Fang , Adv. Mater. 2021, 33, 2103010. 10.1002/adma.202103010 34431141

[8] H. Zhu , A. Liu , K. I. Shim , H. Jung , T. Zou , Y. Reo , H. Kim , J. W. Han , Y. Chen , H. Y. Chu , J. H. Lim , H. J. Kim , S. Bai , Y. Y. Noh , Nat. Commun. 2022, 13, 1741. 35365628 10.1038/s41467-022-29434-x PMC8975846

[9] M. M. Yao , Q. Zhang , D. Wang , R. l. Chen , Y. C. Yin , J. Xia , H. Tang , W. P. Xu , S. H. Yu , Adv. Funct. Mater. 2022, 32, 2202894.

[10] Y. Li , Z. Shi , W. Liang , J. Ma , X. Chen , D. Wu , Y. Tian , X. Li , C. Shan , X. S. Fang , Mater. Horiz. 2021, 8, 1367. 34846447 10.1039/d0mh01567a

[11] J. Yang , W. Kang , Z. Liu , M. Pi , L. B. Luo , C. Li , H. Lin , Z. Luo , J. Du , M. Zhou , X. Tang , J. Phys. Chem. Lett. 2020, 11, 6880. 32627555 10.1021/acs.jpclett.0c01832

[12] M. Zhang , J. Zhu , B. Yang , G. Niu , H. Wu , X. Zhao , L. Yin , T. Jin , X. Liang , J. Tang , Nano Lett. 2021, 21, 1392. 33480701 10.1021/acs.nanolett.0c04197

[13] X. Zhou , L. Zhang , Y. Huang , Z. Zhou , W. Xing , J. Zhang , F. Zhou , D. Zhang , F. Zhao , Adv Opt Mater 2021, 9, 2100889.

[14] Z. Ma , Z. Shi , C. Qin , M. Cui , D. Yang , X. Wang , L. Wang , X. Ji , X. Chen , J. Sun , D. Wu , Y. Zhang , X. J. Li , L. Zhang , C. Shan , ACS Nano 2020, 14, 4475. 32167288 10.1021/acsnano.9b10148

[15] Y. Chen , M. He , J. Peng , Y. Sun , Z. Liang , Adv. Sci. 2016, 3, 1500392. 10.1002/advs.201500392 PMC5069589 27812463

[16] H. P. Wang , S. Li , X. Liu , Z. Shi , X. S. Fang , J. H. He , Adv. Mater. 2021, 33, 2003309. 10.1002/adma.202003309 33346383

[17] A. Feng , X. Jiang , X. Zhang , X. Zheng , W. Zheng , O. F. Mohammed , Z. Chen , O. M. Bakr , Chem. Mater. 2020, 32, 7602.

[18] Z. Q. Li , Z. Li , Z. Shi , X. S. Fang , Adv. Funct. Mater. 2020, 30, 2002634.

[19] X. Mo , T. Li , F. Huang , Z. Li , Y. Zhou , T. Lin , Y. Ouyang , X. Tao , C. Pan , Nano Energy 2021, 81, 105570.

[20] X. Cheng , S. Yang , B. Cao , X. Tao , Z. Chen , Adv. Funct. Mater. 2019, 30, 1905021.

[21] W. Xu , G. Lei , C. Tao , J. Zhang , X. Liu , X. Xu , W.‐Y. Lai , F. Gao , W. Huang , Adv. Funct. Mater. 2018, 28, 1802320.

[22] Y. X. Chen , Q. Q. Ge , Y. Shi , J. Liu , D. J. Xue , J. Y. Ma , J. Ding , H. J. Yan , J. S. Hu , L. J. Wan , J. Am. Chem. Soc. 2016, 138, 16196. 27998083 10.1021/jacs.6b09388

[23] Y. Liu , Y. Zhang , Z. Yang , D. Yang , X. Ren , L. Pang , S. F. Liu , Adv. Mater. 2016, 28, 9204. 27569400 10.1002/adma.201601995

[24] Z. Q. Li , C. Zuo , X. Liu , Z. Ma , Z. Shi , X. S. Fang , Adv. Opt. Mater. 2021, 10, 2102315.

[25] Y. Liu , Y. Zhang , Z. Yang , J. Feng , Z. Xu , Q. Li , M. Hu , H. Ye , X. Zhang , M. Liu , K. Zhao , S. Liu , Mater. Today 2019, 22, 67.

[26] V. K. LaMer , R. H. Dinegar , J. Am. Chem. Soc. 1950, 72, 11.

[27] L. Wei , J. Yao , H. Fu , ACS Nano 2013, 7, 7573. 23937335 10.1021/nn402889h

[28] X. Zhao , T. Liu , Y. Cui , X. Hou , Z. Liu , X. Dai , J. Kong , W. Shi , T. J. S. Dennis , Nanoscale 2018, 10, 8170. 29676419 10.1039/c8nr01305e

[29] Y. Liu , Z. Yang , D. Cui , X. Ren , J. Sun , X. Liu , J. Zhang , Q. Wei , H. Fan , F. Yu , X. Zhang , C. Zhao , S. F. Liu , Adv. Mater. 2015, 27, 5176. 26247401 10.1002/adma.201502597

[30] Z. Ma , Z. Shi , D. Yang , Y. Li , F. Zhang , L. Wang , X. Chen , D. Wu , Y. Tian , Y. Zhang , L. Zhang , X. Li , C. Shan , Adv. Mater. 2021, 33, 2001367. 10.1002/adma.202001367 33225543

[31] Q. Dong , Y. Fang , Y. Shao , P. Mulligan , J. Qiu , L. Cao , J. Huang , Science 2015, 347, 6225. 10.1126/science.aaa5760 25636799

[32] W. G. Li , X. D. Wang , J. F. Liao , Y. Jiang , D. B. Kuang , Adv. Funct. Mater. 2020, 30, 1909701.

[33] X. Xing , T. Tong , M. Mohebinia , D. Wang , Z. Ren , V. G. Hadjiev , Z. Wang , J. Bao , J. Phys. Chem. Lett. 2022, 13, 6447. 35816284 10.1021/acs.jpclett.2c01544

[34] S. Zhao , W. Cai , H. Wang , Z. Zang , J. Chen , Small Methods 2021, 5, 2001308. 10.1002/smtd.202001308 34928084

[35] F. Cao , L. Li , Adv. Funct. Mater. 2020, 31, 2008275.

[36] Z. Guo , J. Li , R. Pan , J. Cheng , R. Chen , T. He , Nanoscale 2020, 12, 15560. 32692791 10.1039/d0nr04220j

