# Supplementary material for: Anisotropic Growth of Centimeter‐Size CsCu2I3 Single Crystals with Ultra‐Low Trap Density for Aspect‐Ratio‐Dependent Photodetectors
Source: Adv Sci (Weinh). 2023 Jan 4;10(7):2206417. doi: 10.1002/advs.202206417 (PMC9982547; doi:10.1002/advs.202206417)
Supplement: Supplementary file 1 — Supporting Information [file ADVS-10-2206417-s001.pdf]

Supporting Information

Experimental Section

Synthesis of $CsCu_2I_3$ single crystal

$CsCu_2I_3$ single crystal was grown by following a modified low-cost antisolvent vapor assisted method, the detailed process is depicted as follows. The raw materials CsI (Aladdin Chemistry Co., Ltd.,99.9%), CuI (Aladdin Chemistry Co., Ltd., 99.5%), dimethyl sulfoxide (J&K Scientific Ltd., DMSO, 99.7%), and N,N-dimethylformamide (J&K Scientific Ltd., DMF, 99.8%) were used during the growth process. In the investigation of the effect of molar ratio on the mass of single crystals, CsI and CuI were homogeneously mixed and dissolved in 2 ml of solvent mixture (DMSO: DMF = 1:1) at different molar ratios (1:1, 1:2, 3:2, 3:8), respectively. After stirring the mixture at 60 °C for 2 h, the precursor solution was directly centrifuged at 8000 rpm for 5 min. The saturated supernatant was injected in a vial and covered with a paraffin film with a small hole. This vial was placed in a large vial, which contained methanol anhydrous and was finally sealed by paraffin film. The whole device was placed in an oil bath at 60 °C for 36 h. The grown single crystals were obtained and rinsed with isopropanol and ethanol. In the investigation of the effect of different solvents on the mass of single crystals, a mixture of CsI and CuI with a molar ratio of 3:2 was dissolved in DMF/DMSO (1:1), DMF/ME (1:1), and DMSO/ME (1:1), both in 2 ml, respectively, and all other experimental conditions

were the same as above. When investigating the effect of growth time on single crystals, multiple devices for growing single crystals as shown above were prepared and each device was ensured to have the same parameters between them (where CsI:CuI=3:2; solvent is DMF/DMSO). Each device was started simultaneously in a correspondingly different oil bath, ensuring that all parameters and experimental operations were identical except for the growth time. In studying the effect of growth temperature on the quality of single crystals, other growth parameters were ensured to be constant (CsI:CuI= 3:2; DMF / DMSO solvent; growth time of 36h), and temperature gradients (40°C, 60°C, 80°C,,100°C) were set for the growth of single crystals. In the study of the effect of the velocity of the anti-solvent vapor into the precursor solution on the single crystals, several precursor devices with different small pores were prepared and then all of them were placed in the same large vial containing methanol, while other experimental operations were kept constant. (where CsI:CuI=3:2; solvent is DMF/DMSO; Growth time is 36h). When studying the effect of solvent concentration on single crystal size, the other growth parameters are guaranteed to be constant and the concentration gradient is set for single crystal growth.

Characterizations:

The phase was identified by XRD (Bruker D8-A25) with CuK radiation (λ = 0.15406 nm). The powder sample was tested with the scan step of 0.02° and a step time of 1 s. The optical photographs were obtained by Olympus optical microscope.

The morphology of $CsCu_2I_3$ single crystal was traced by field emission scanning electron microscope (FESEM) (Zeiss Sigma). The FIB (Helios G4) is carried out to cut the slices for TEM (SPECTRA 300S) characterization. X-ray photoelectron spectra were acquired by the PHI 5000C&PHI5300 X-ray photoelectron spectrometer, equipped with a dual Mg/Al anode. All peaks were calibrated using the C 1s peak (284.6 eV) as the reference. The steady-state photoluminescence (PL) spectrum was performed by Horiba Fluorolog-3 instrument. The transient PL spectrum was carried out using a fluorescence lifetime spectrometer with a pulsed nano-LED excitation source. The optical absorption spectra were investigated by UV-vis spectrophotometer (Hitachi, U-3900H). The light intensity was measured with a NOVA II power meter (OPHIR photonics). The photoelectric properties of the PDs were recorded using the semiconductor characterization system (Keithley 4200-SINGLE CRYSTALS), connected to a 75 W xenon arc lamp with a monochromator. The light intensity was determined with a NOVA II power meter (OPHIR Photonics). All the measurements were performed at room temperature.

First principles calculations:

All calculations in this study were performed with the Vienna ab initio Simulation Package (VASP) within the frame of density functional theory (DFT) [1]. The exchange-correlation interactions of electron were described via the generalized gradient approximation (GGA) with PBE functional [2], and the projector augmented wave (PAW) method [3] was used to describe the interactions of electron and ion.

Additionally, the DFT-D3 method [4,5] was used to account for the long-range van der Waals forces present within the system. The Monkhorst-Pack scheme [6] with a 2x2x1

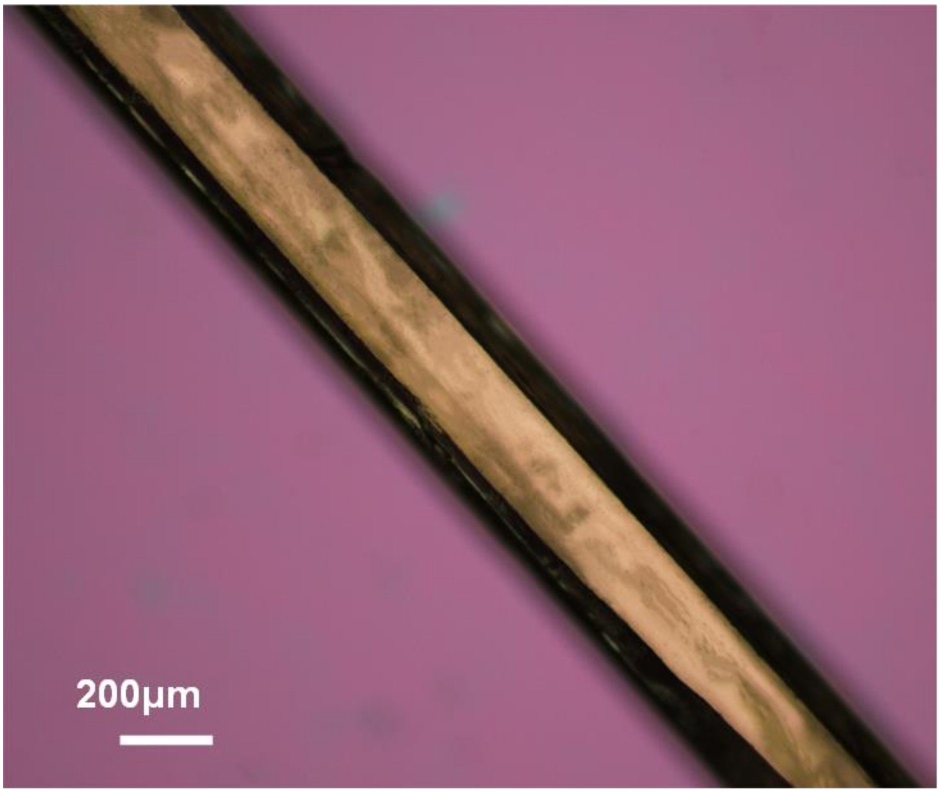

k-point mesh was used for the integration in the irreducible Brillouin zone. The kinetic energy cut-off of 500 eV was chosen for the plane wave expansion. The lattice parameters and ionic position were fully relaxed, and the total energy was converged within 10-5 eV per formula unit. The final forces on all ions are less than 0.02/Å.

Figure S1. Light microscope photograph of a single crystal.

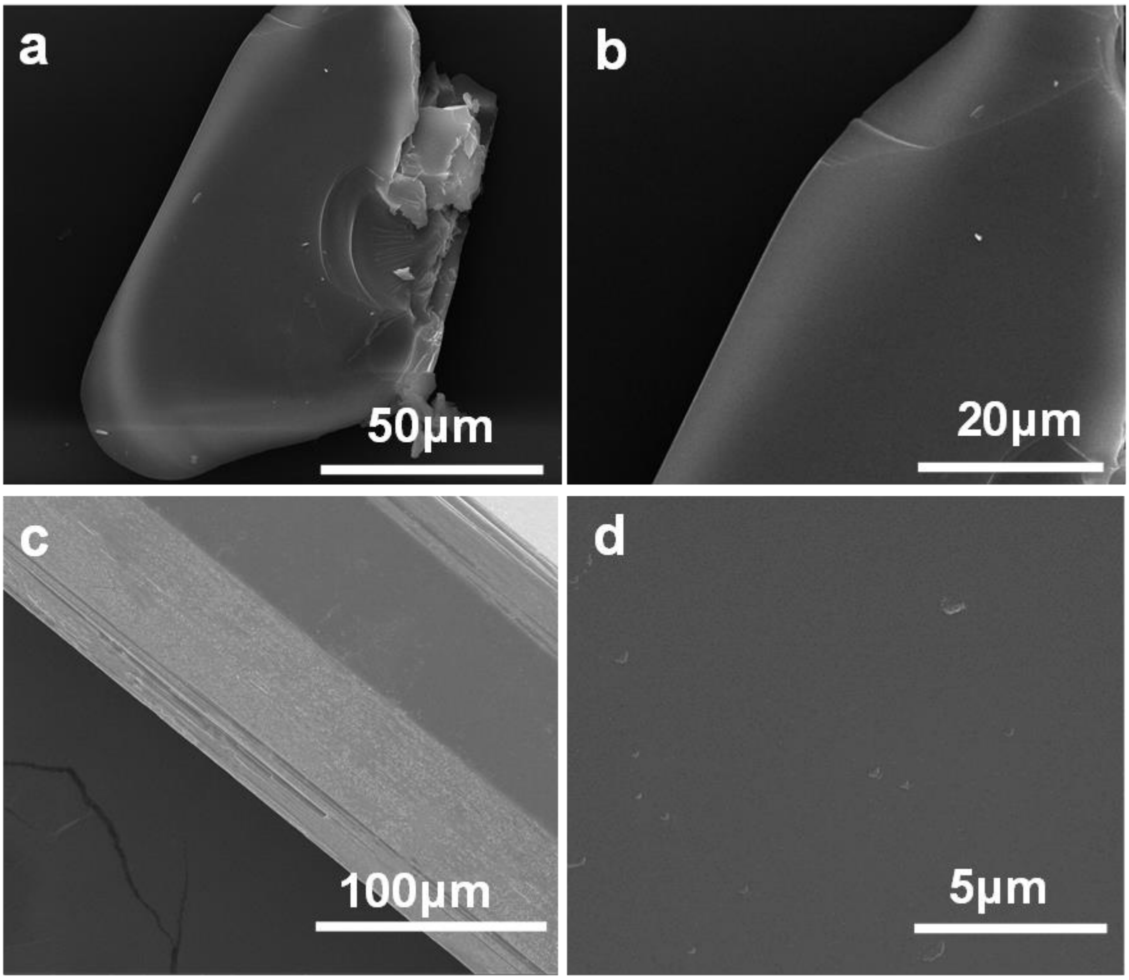

Figure S2. The SEM image of $CsCu_2I_3$ crystals plate prepared by ultrasonic treatment and Centrifugal process from bulk crystals: (a) The scale bar is 50 μm (b) The scale bar is 50 μm; The SEM image of $CsCu_2I_3$ bulk crystals (c) The scale bar is 100 μm; (d) The scale bar is 5μm.

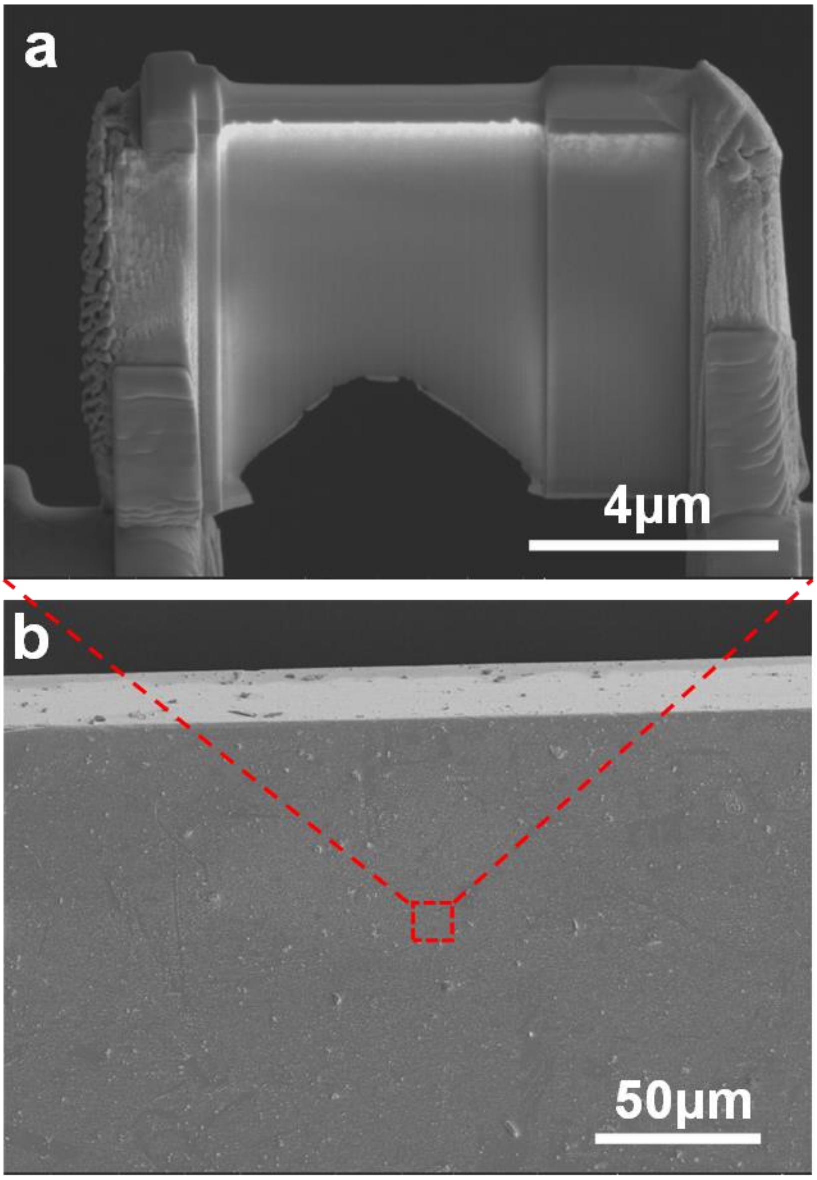

Figure S3. $CsCu_2I_3$ single crystal FIB slice images: (a) FIB slice image of $CsCu_2I_3$ single crystal in a metal frame (b) image of the cut single crystal (the red box is marked as the cut position of the FIB slice)

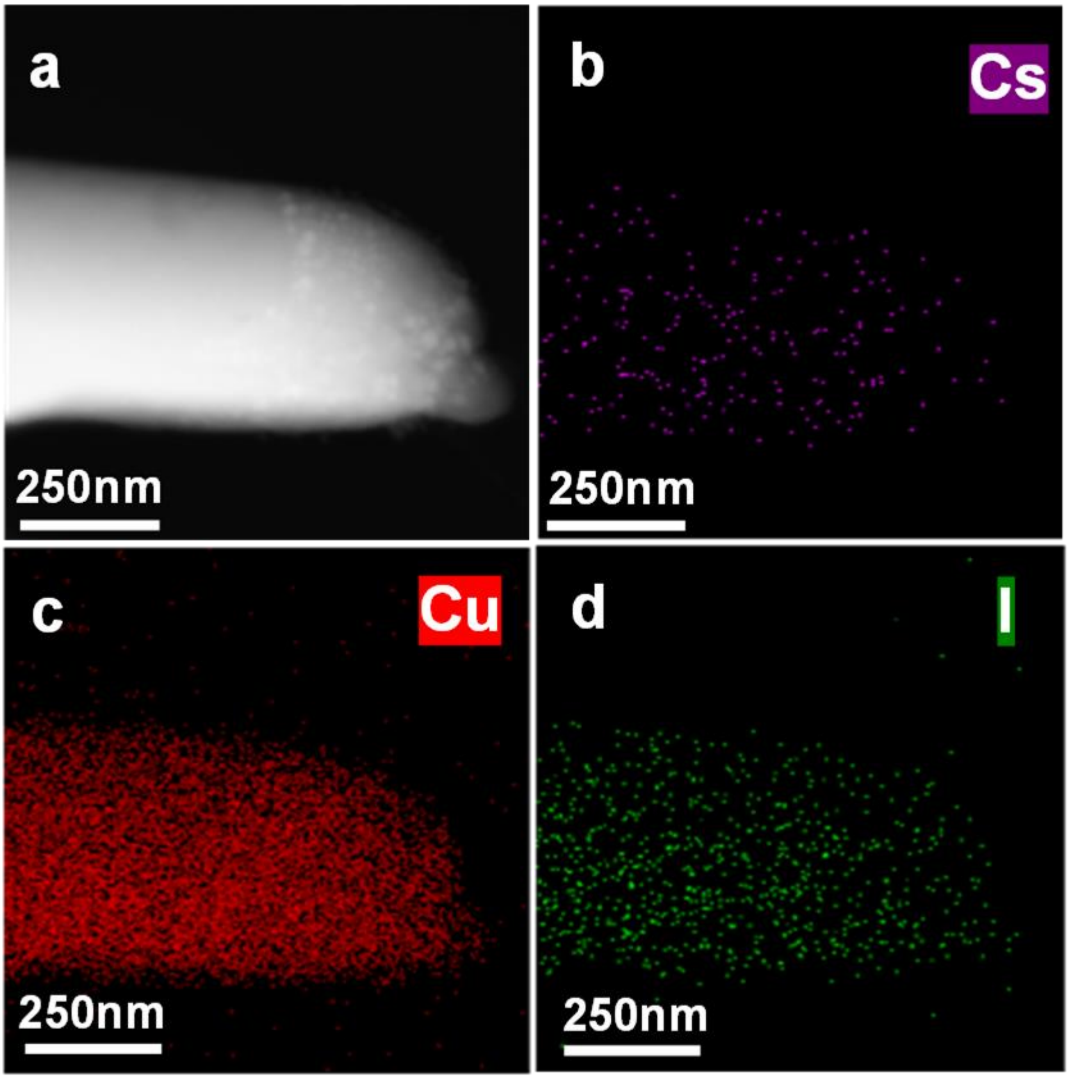

Figure S4. Elemental mapping images of $CsCu_2I_3$

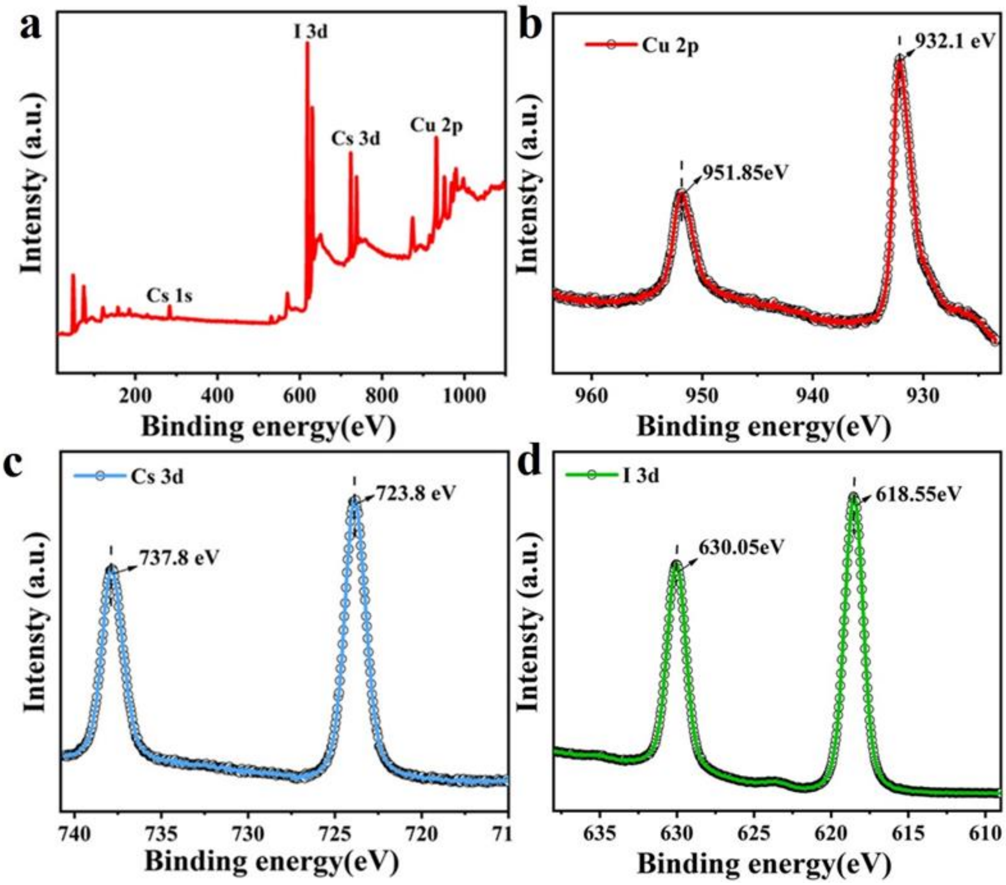

Figure S5. (a) survey spectrum and high-resolution X-ray photoelectron spectra (b) Cu 2p core level (c)Cs 3d core level, (d) I 3d core level of ultra-long, high aspect ratio centimeter-scale CsCu2I3 single crystal.

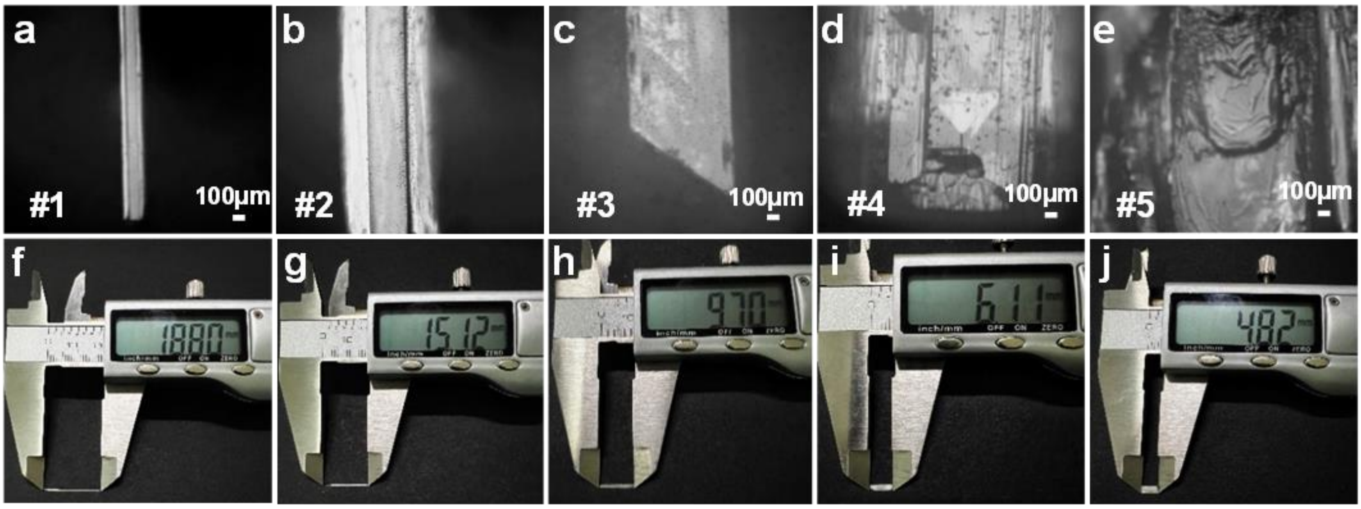

Figure S6. Photographs of single crystals with different sizes affected by different anti-solvent vapor diffusion rates: (a-e) Optical microscope photo of the single crystal. The scale bar is 100 µm. (f-j) Vernier caliper test single crystal length.

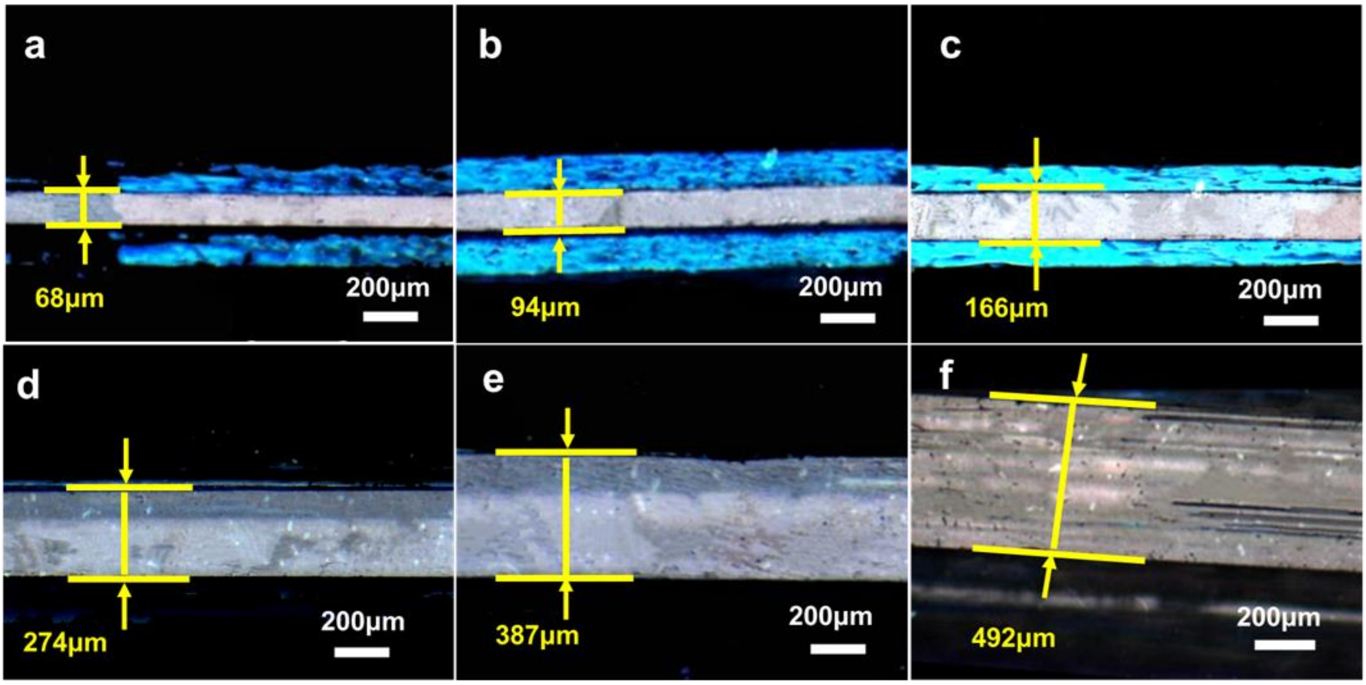

Figure S7. Optical micrographs of single crystals of different sizes affected by the concentration of precursor solution. The scale bar is 200 μm.

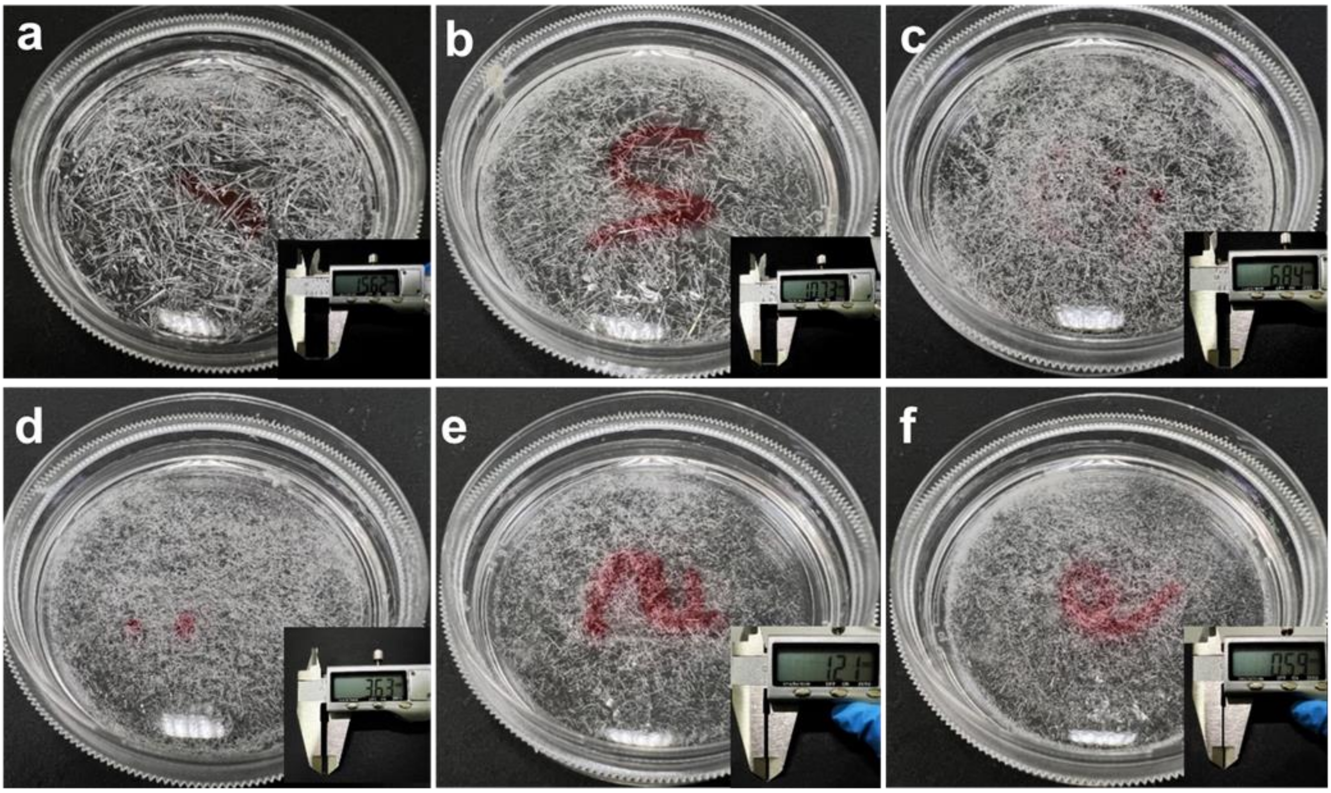

Figure S8. Photographs of fresh single crystals prepared at different precursor solution concentrations (inset is a photograph of the single crystal measured with vernier calipers) **a**.2.5mol/L, **b**.1.25mol/L, **c**.0.833mol/L, **d**.0.625mol/L, **e**.0.5mol/L, **f**. 0.417mol/L

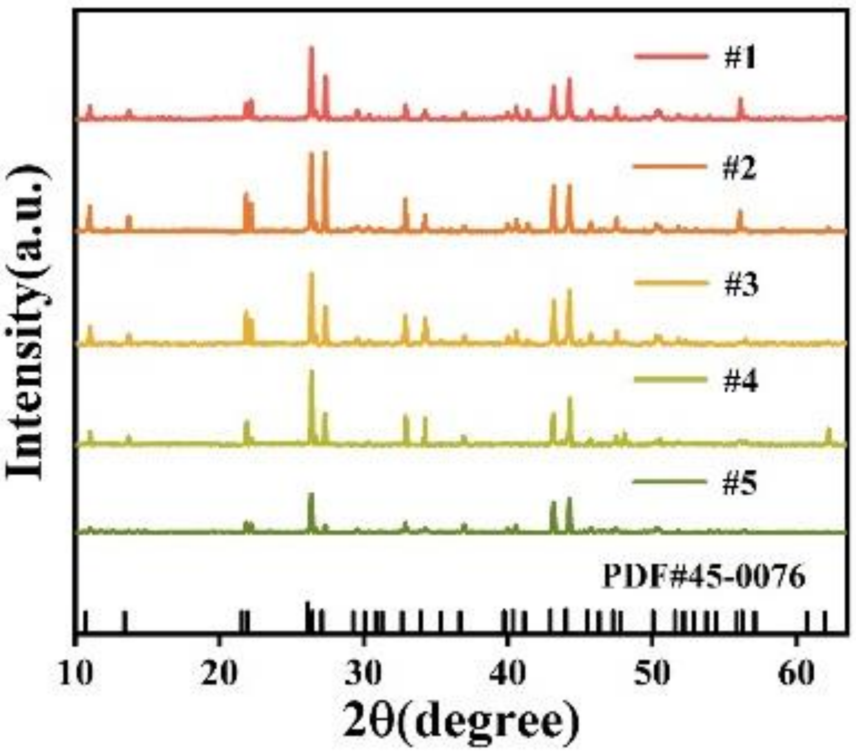

Figure S9. XRD pattern of the as-grown single crystals prepared with different diffusion rate of antisolvent vapor in precursor solution.

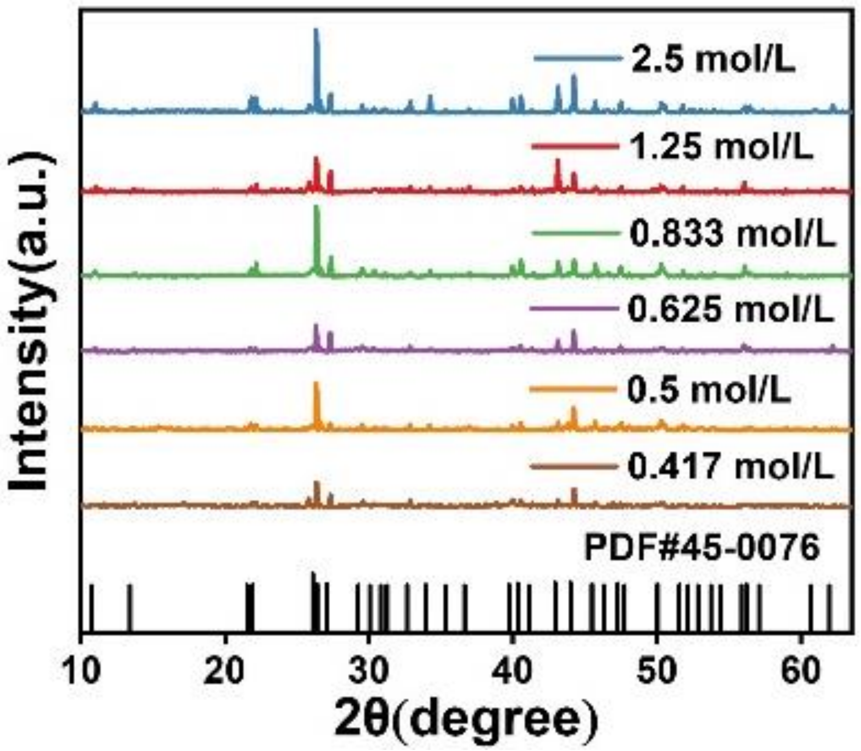

Figure S10. XRD pattern of the as-grown single crystals prepared different precursor solution concentrations.

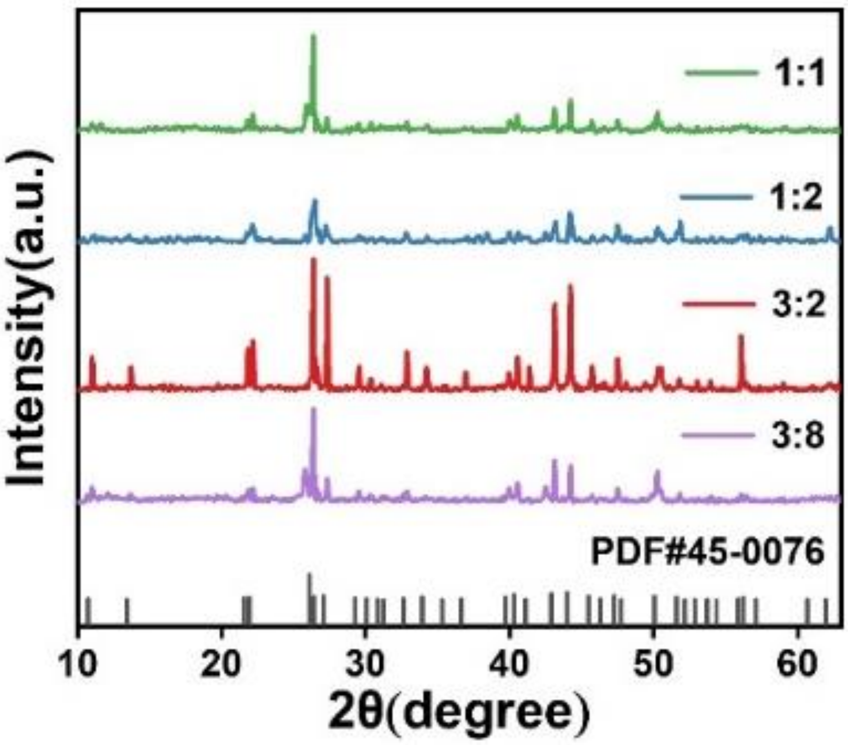

Figure S11. XRD pattern of the as-grown single crystals prepared different mole ratio of reagents.

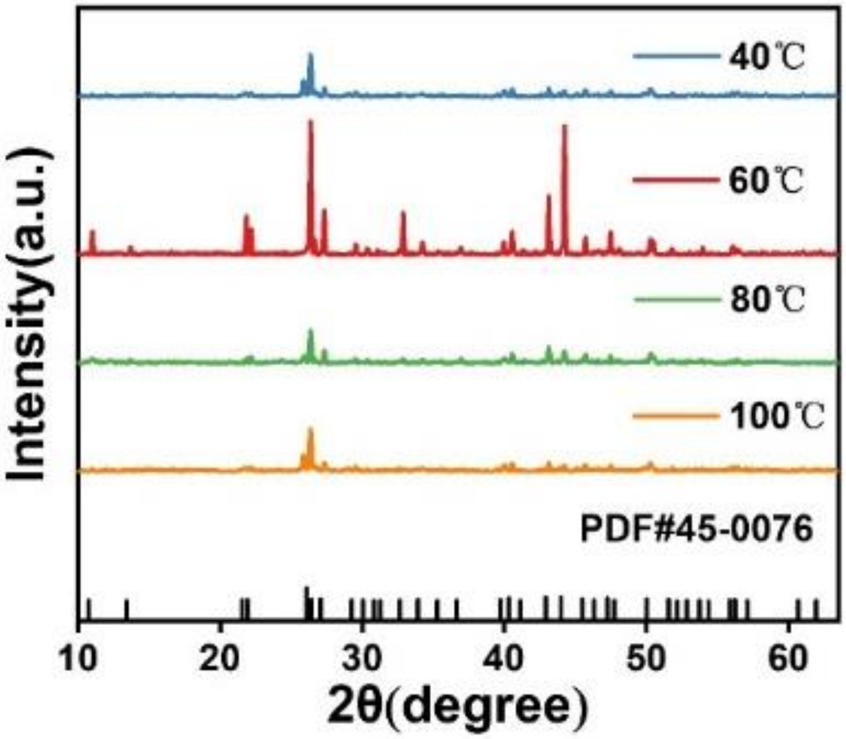

Figure S12. XRD pattern of the as-grown single crystals prepared with different growing temperature.

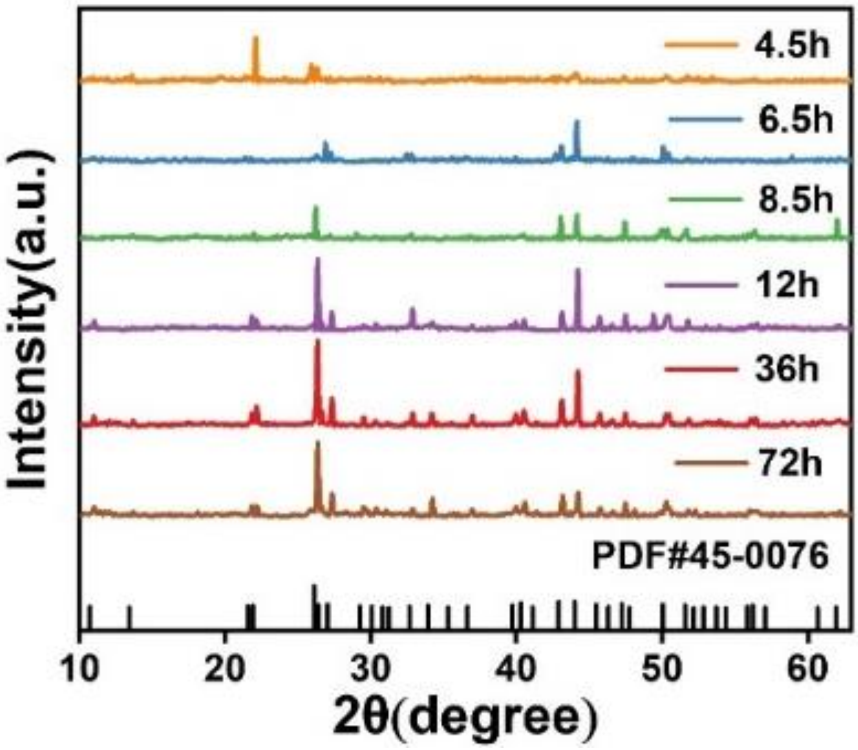

Figure S13. XRD pattern of the as-grown single crystals prepared with different growing time.

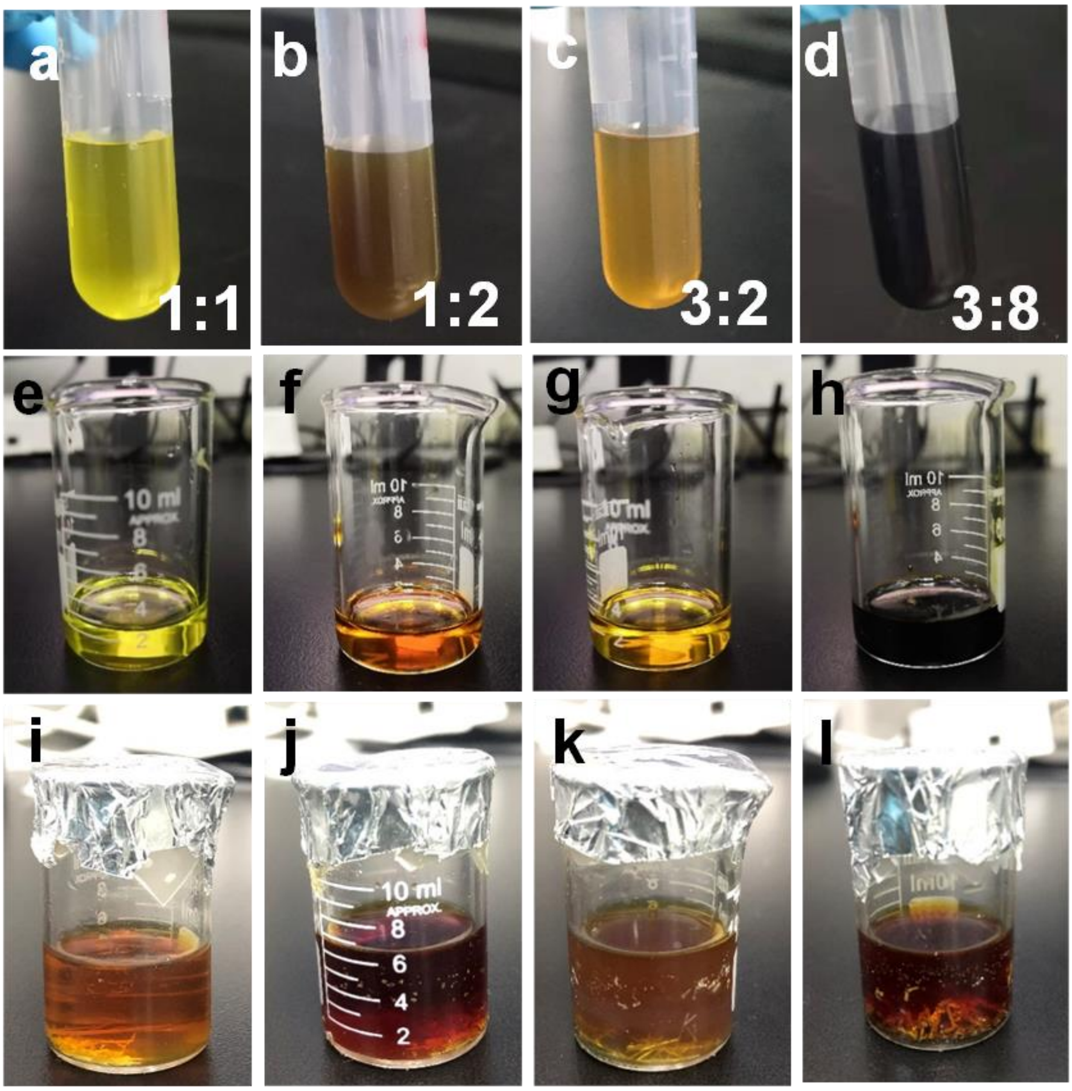

Figure S14. The photographs of the precursor solutions at different stages of synthesis process. (a-d) After stirring. (e-f) Saturated supernatant after centrifugation. (c) At the end of growth process.

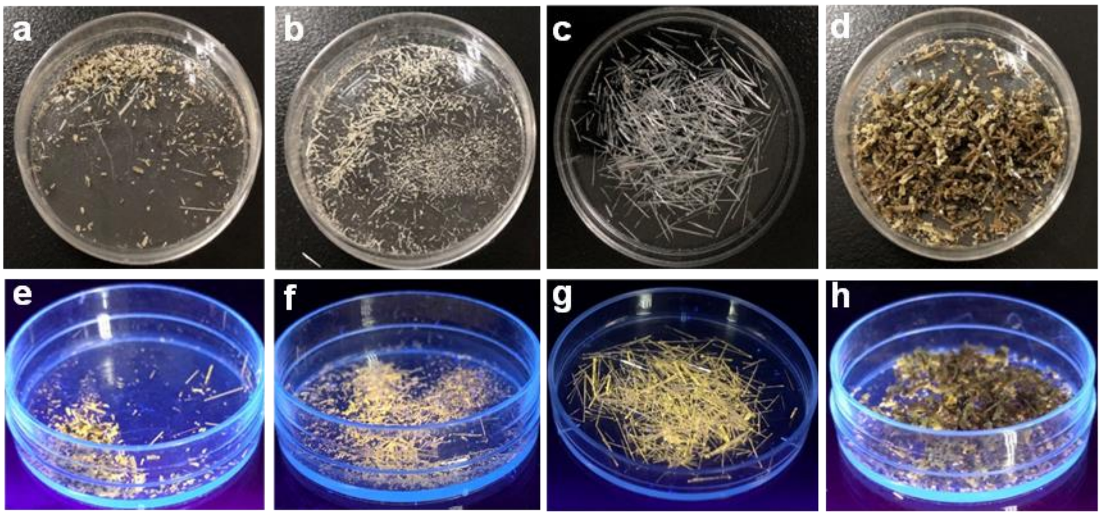

Figure S15. (a-d) Photographs of single crystals grown at different molar ratios. (e-h) Photographs of single crystal luminescence grown at different molar ratios. (a)(e) CsI:CuI=1:1; (b)(f) CsI:CuI=1:2; (c)(g) CsI:CuI=3:2; (d)(h) CsI:CuI=3:8.

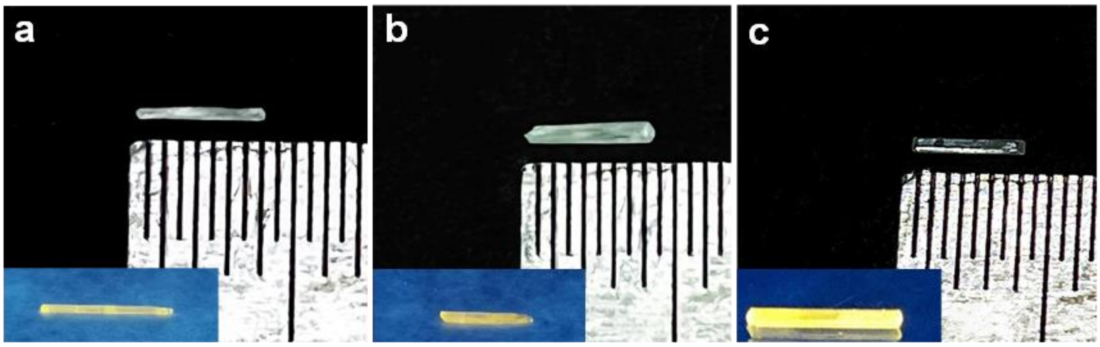

Figure S16. $CsCu_2I_3$ single crystals prepared under different solvents(a)DMF/DMSO, (b) DMF/ME, (c) DMSO/ME

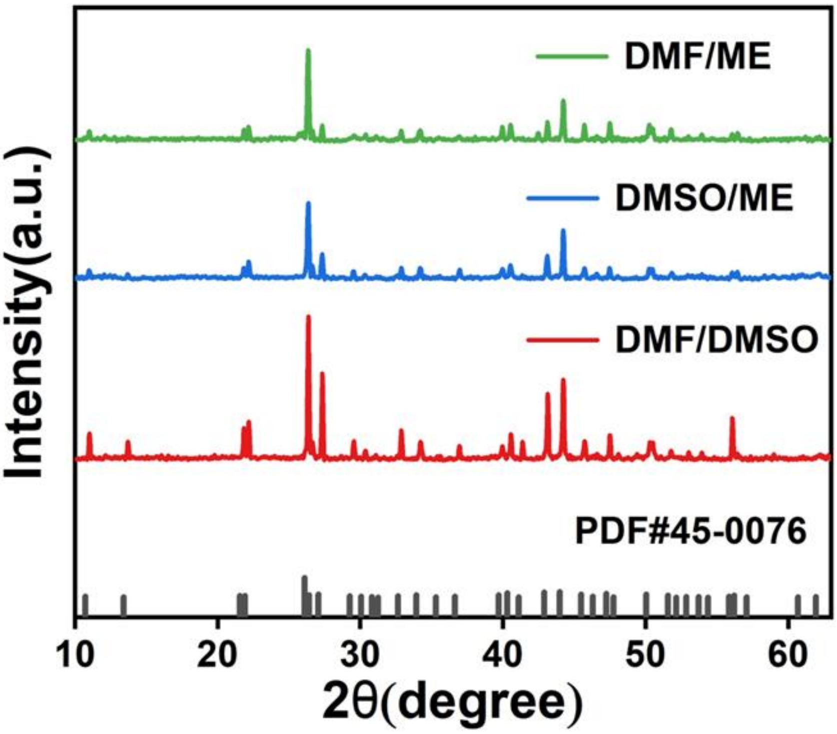

Figure S17. Powder X-ray diffraction pattern of the as-grown single crystals prepared with different solvents（a）DMF/DMSO, (b) DMF/ME, (c) DMSO/ME.

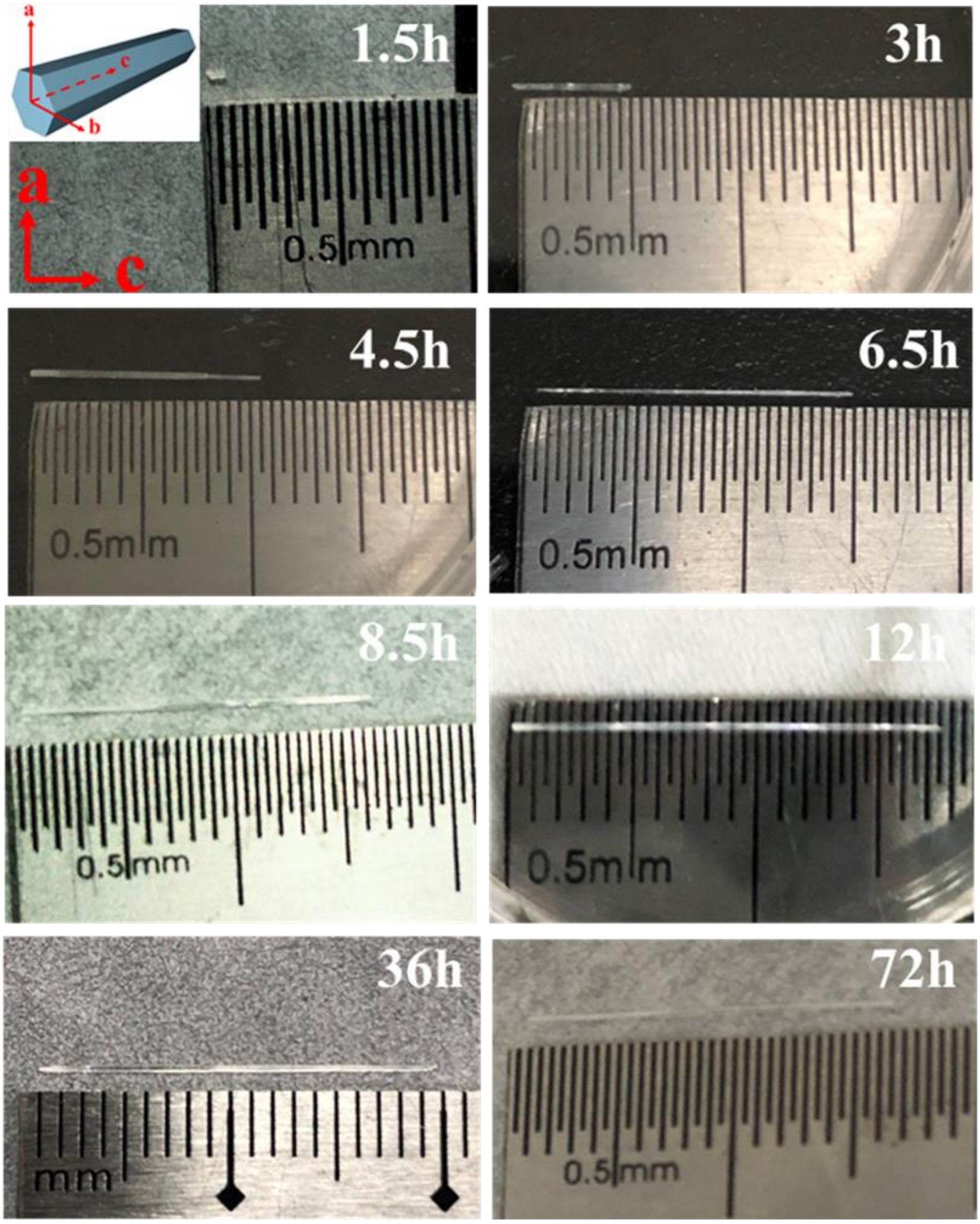

Figure S18. Real-time growth observations of $CsCu_2I_3$ single crystals (Inset is schematic view of the corresponding crystal morphology) .

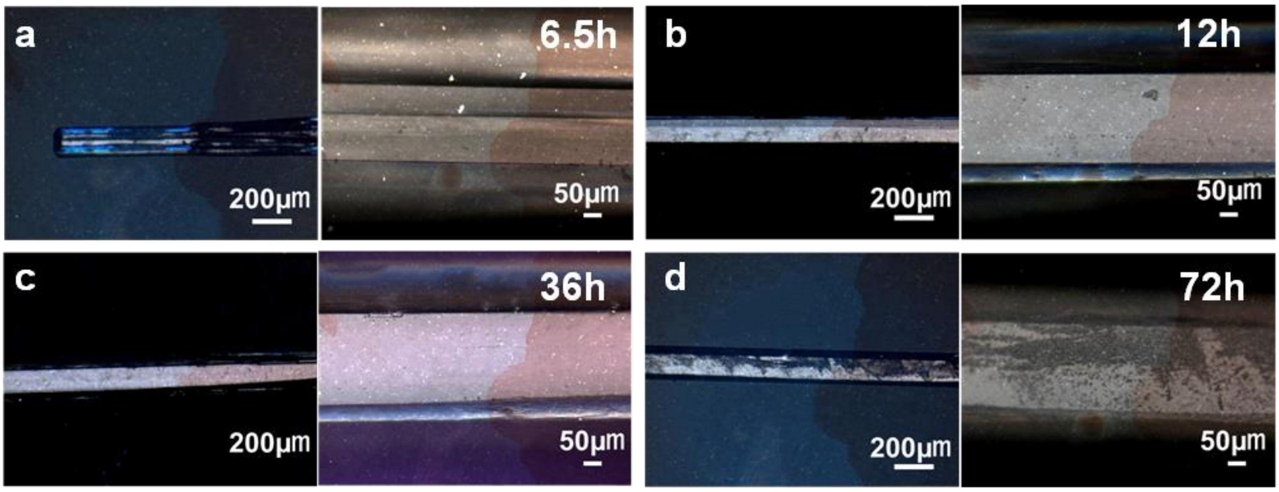

Figure S19. Polarized light micrographs of $CsCu_2I_3$ single crystals at different growth times. (a-d) Growth duration was 6.5h, 12h, 36h, 72h, respectively.

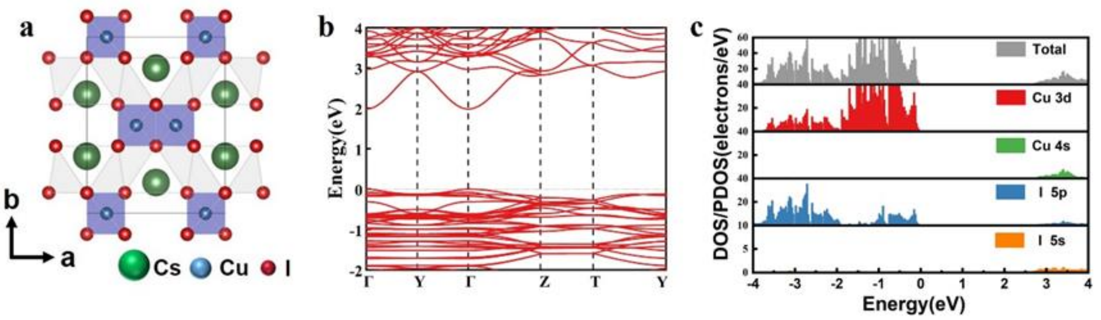

Figure S20. (a) 1D crystal structure of $CsCu_2I_3$ single crystal: Perspective view of crystal structure along [001] direction (c-axis); (b) Calculated electronic band structure of the 1D $CsCu_2I_3$; (c)Total and partial electronic DOS for the 1D $CsCu_2I_3$.

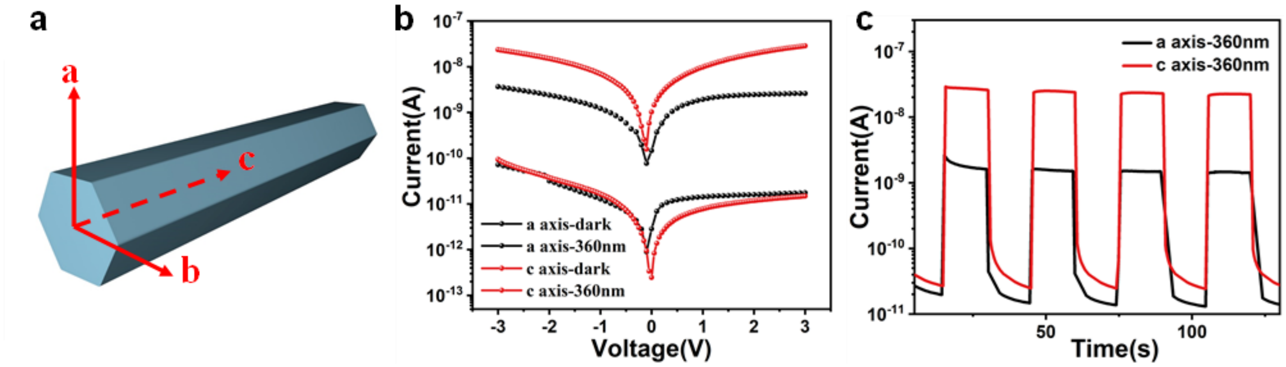

Figure S21. Comparison of photoelectric properties between the crystallographic c direction and the crystallographic **a** direction of CsCu2I3 single crystals: a Schematic view of the corresponding crystal morphology. **b** I–V curves under dark and 360 nm illumination at 3 V bias. **c** I–T curves.

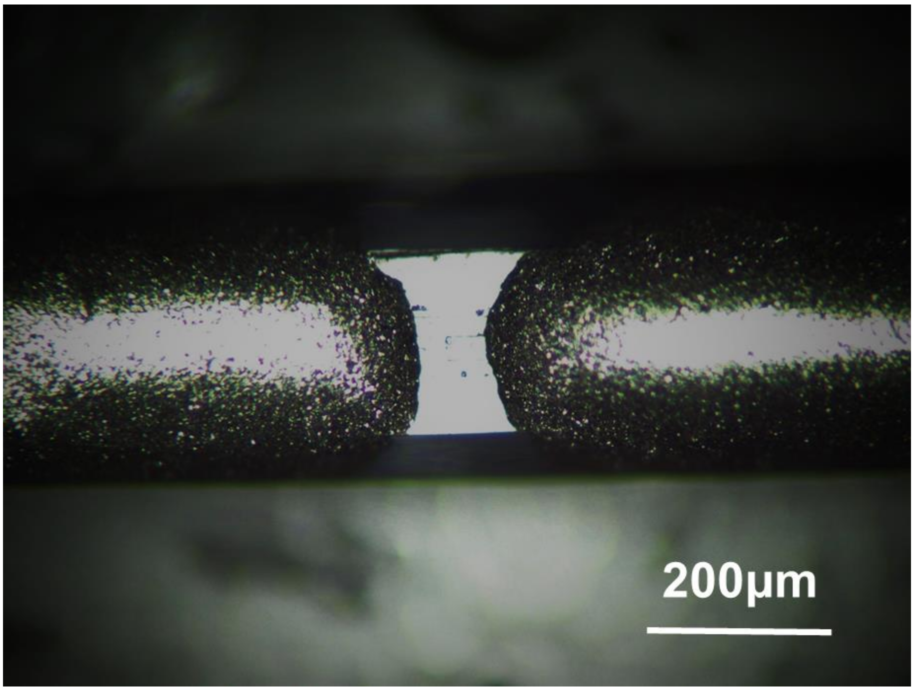

Figure S22. Photograph of the device.

Table S1 Size statistics of prepared single crystals at different antisolvent vapor diffusion rates

Number	#1 (1 small hole)	#2 (3 small hole)	#3 (6 small hole)	#4 (9 small hole)	#5 (12 small hole)
Length(mm)	4.82±0.82	6.11±0.54	9.7±0.48	15.12±0.46	18.80±0.33
Diameter(μm)	2590±180	1526±124	842±176	305±32	158±45

Table S2 Size statistics of prepared single crystals at different precursor solution concentrations

Number	A (2.5 mol/L)	B (1.25 mol/L)	C (0.833 mol/L)	D (0.625 mol/L)	E (0.5 mol/L)	F (0.417 mol/L)
Length(mm)	15.62±1.38	10.73±1.84	6.84±1.62	3.63±0.76	1.21±0.46	0.59±0.32
Diameter(μm)	353±32	287±28	197±26	119±18	89±20	72±13

Table S3. Summaries of performance of UV photodetectors based on copper-based perovskites and other typical all-inorganic metal halide perovskites.

Material structure	Response range (nm)	Bias (V)	On-off ratio	Responsivity (mA W^{-1})	D* ($\times10^{12}$ jones)	EQE(%)	Reference
Ag–$CsCu_2I_3$ SC–Ag	360	3	1570	277	1.34	96	This work
Ag–$CsCu_2I_3$ SC–Ag	350	3	31.5	51	0.092	19	[7]
Ni–$CsCu_2I_3$ SC –Ni	340	-5	4	——	——	——	[8]
Au/$CsCu_2I_3$ films/Au	340	2	13.7	0.66	0.018	——	[9]
Au/$CsCu_2I_3$/CuI films/Au	340	2	3150	49	2.49	——	[9]
Ag–$CsCu_2I_3$ SC-Ag	350	3	600	126	0.64	——	[10]
Ag–$CsCu_2I_3$ SC/CuI–Au	350	3	280	253	0.31	90	[10]
Au–$CsCu_2I_3$ microbelt-Au	300	0	20	0.014	0.0025	——	[10]
Au-$CsCu_2I_3$ films/GaN-In	300	0	1×10^5	47.63	2.58	21.5	[11]
Au-$CsCu_2I_3$ Arrays-Au	365	-5	1121	80	2.7	27	[12]
LiF -$CsCu_2I_3$ films/Si-Au	330	-1	590	140	0.37	——	[13]
Au-CuI/ $CsCu_2I_3$ /GaN-In	365	0	97886	71.7	3.3	26.1	[14]
Au-$CsCu_2I_3$ Film-Au	265	3	22	22.1	0.12	10.3	[15]
ITO-$Cs_3Bi_2I_9$ SC-Au	White light	-2	11000	7.2	0.0093	——	[16]
ITO-$Cs_3Bi_2Br_9$ SC -ITO	400	10	——	0.025	0.0008	——	[17]
Au-$Cs_2SnCl_{6-x}Br_x$ SC-Au	590	20	——	0.0271	——	——	[18]
Ag-$Cs_2AgBiBr_6$ Sc-Ag	400	5	——	0.9	0.0026	——	[19]
Au/$Cs_2AgInCl_6$ Sc /Au	365	5	——	13	0.96	——	[20]
Au/$CsAg_2I_3$ Sc /Au	265	2	——	0.355	0.00295	0.17	[21]
Au/Cr–Zn_2GeO_4–Au/Cr	254	20	——	——	——	——	[22]
Au–CsPb 2 Br 3 –Au	254	2	1000	0.24	0.01		[23]

References:

[1] G. Kresse, J. Furthmuller, *Phys. Rev. B*. **1996**, *54*, 16.

[2] J.P. Perdew, K. Burke, M. Ernzerhof, *Phys. Rev. Lett*. **1996**, *77*, 3865.

[3] G. Kresse, J. Joubert, *Phys. Rev. B*. **1999**, *59*, 1758.

[4] S. Grimme, J. Antony, S. Ehrlich, H. Krieg, *J. Chem. Phys*. **2010**, *132*, 154104.

[5] S. Grimme, S. Ehrlich, L. Goerigk, *J. Comput. Chem*. **2011**, *32*, 1456.

[6] H.J. Monkhorst, J.D. Pack, *Phys. Rev. B*. **1976**, *13*, 5188.

[7] Z. Q. Li, Z. Li, Z. Shi, X. S. Fang, *Adv. Funct. Mater.* **2020**, *30*, 2002634.

[8] X. Mo, T. Li, F. Huang, Z. Li, Y. Zhou, T. Lin, Y. Ouyang, X. Tao, C. Pan, *Nano Energy* **2021**, *81*, 105570.

[9] X. Zhou, L. Zhang, Y. Huang, Z. Zhou, W. Xing, J. Zhang, F. Zhou, D. Zhang, F. Zhao, *Adv. Opt. Mater.* **2021**, *9*, 2100889.

[10] Z. Q. Li, C. Zuo, X. Liu, Z. Ma, Z. Shi, X. S. Fang, *Adv. Opt. Mater.* **2021**, *10*, 2102315

[11] M. Li, C. Cao, W. Liu, N. Wang, C. Yi, R. Li, J. Wang, *J. Phys. Chem. Lett.* **2022**, *13*, 6462.

[12] X. Xu, S. Jiang, C. Fan, Q. Deng, L. Shen, Q. Zhang, *Adv. Opt. Mater.* **2022**, *10*, 2201107.

[13] C. Wang, F. Zhao, Z. Zhou, X. Li, S. He, M. Zhang, D. Zhang, L. Zhang, *J. Alloy. Compd.* **2022**, *905*, 164245.

[14] X. Zhou, C. Wang, J. Luo, L. Zhang, F. Zhao, Q. Ke, *Chem. Eng. J.* **2022**, *450*, 136364.

[15] J. Yang, W. Kang, Z. Liu, M. Pi, L. B. Luo, C. Li, H. Lin, Z. Luo, J. Du, M. Zhou, X. Tang, *J. Phys. Chem. Lett.* **2020**, *11*, 6880.

[16] W. G. Li, X. D. Wang, J. F. Liao, Y. Jiang, D. B. Kuang, *Adv. Funct. Mater.* **2020**, *30*, 1909701.

[17] Y. Tang, M. Liang, B. Chang, H. Sun, K. Zheng, T. Pullerits, Q. Chi, *J. Mater. Chem.C* **2019**, *7*, 3369.

[18] J. Zhou, J. Luo, X. Rong, P. Wei, M. S. Molokeev, Y. Huang, J. Zhao, Q. Liu, X.

Zhang, J. Tang, Z. Xia, *Adv. Opt. Mater.* **2019**, *7*, 1900139.

[19] Y. Dang, G. Tong, W. Song, Z. Liu, L. Qiu, L. K. Ono, Y. Qi, *J. Mater. Chem. C* **2020**, *8*, 276.

[20] J. Luo, S. Li, H. Wu, Y. Zhou, Y. Li, J. Liu, J. Li, K. Li, F. Yi, G. Niu, J. Tang, *ACS Photonics* **2017**, *5*, 398.

[21] M. M. Yao, Q. Zhang, D. Wang, R. l. Chen, Y. C. Yin, J. Xia, H. Tang, W. P. Xu, S. H. Yu, *Adv. Funct. Mater.* **2022**, *32*, 2202894.

[22] C. Yan, N. Singh, P. S. Lee, *Appl. Phys. Lett.* **2010**, *96*, 053108.

[23] T. Zhang, F. Wang, P. Zhang, Y. Wang, H. Chen, J. Li, J. Wu, L. Chen, Z. D. Chen, S. Li, *Nanoscale* **2019**, *11*, 2871.
